# Optimal synaptic signaling connectome for locomotory behavior in *Caenorhabditis elegans*: Design minimizing energy cost

**DOI:** 10.1371/journal.pcbi.1005834

**Published:** 2017-11-20

**Authors:** Franciszek Rakowski, Jan Karbowski

**Affiliations:** 1 Interdisciplinary Centre for Mathematical and Computational Modeling, University of Warsaw, Warsaw, Poland; 2 Mossakowski Medical Research Centre, Polish Academy of Sciences, Warsaw, Poland; 3 Nalecz Institute of Biocybernetics and Biomedical Engineering, Polish Academy of Sciences, Warsaw, Poland; 4 Institute of Applied Mathematics and Mechanics, Department of Mathematics, Informatics and Mechanics, University of Warsaw, Warsaw, Poland; Hamburg University, GERMANY

## Abstract

The detailed knowledge of *C. elegans* connectome for 3 decades has not contributed dramatically to our understanding of worm’s behavior. One of main reasons for this situation has been the lack of data on the type of synaptic signaling between particular neurons in the worm’s connectome. The aim of this study was to determine synaptic polarities for each connection in a small pre-motor circuit controlling locomotion. Even in this compact network of just 7 neurons the space of all possible patterns of connection types (excitation vs. inhibition) is huge. To deal effectively with this combinatorial problem we devised a novel and relatively fast technique based on genetic algorithms and large-scale parallel computations, which we combined with detailed neurophysiological modeling of interneuron dynamics and compared the theory to the available behavioral data. As a result of these massive computations, we found that the optimal connectivity pattern that matches the best locomotory data is the one in which all interneuron connections are inhibitory, even those terminating on motor neurons. This finding is consistent with recent experimental data on cholinergic signaling in *C. elegans*, and it suggests that the system controlling locomotion is designed to save metabolic energy. Moreover, this result provides a solid basis for a more realistic modeling of neural control in these worms, and our novel powerful computational technique can in principle be applied (possibly with some modifications) to other small-scale functional circuits in *C. elegans*.

## Author summary

Neural connectomes, i.e. neural connectivity maps, are important for understanding the design principles of nervous systems. However, they are not sufficient for understanding network dynamics, which in turn are related to animal’s behavior. To understand behavior, we need additionally to know the type of signaling mediated by neural connections, or simply their polarities (excitation or inhibition). But the determination of these polarities is generally challenging because of the large number of synapses in a typical network. The small nematode *Caenorhabditis elegans* with 302 neurons is the only animal with the known connectome on a level of single neurons. In this study, we use a powerful and fast computational technique to optimize intrinsic properties of *C. elegans* network controlling locomotion in order to determine the signaling signs of its connections. As a result, we find that all locomotory interneuron connections are inhibitory, which suggests that this network acts mainly via mutual suppression.

## Introduction

Neural connectomes of different animals posses interesting topological features such as hierarchy, motifs, and invariants, which are shared across brains with different size, from nematode worms to mammals [1, 2, 3, 4, 5, 6, 7, 8]. However, neither the knowledge of macroscopic [2] nor microscopic [5, 6, 8] connectivity of brain networks is sufficient to predict their dynamics or animal behavior. Functional properties of neural networks, apart from the connectome, depend also on other characteristics such as the type of synaptic transmission, its modulation, incoming input, etc [9, 10, 11].

The fundamental problem in neuroscience, and a big obstacle in modeling behavior, is that we do not know simultaneously the detailed microscopic connectome and synaptic signaling (putting the input aside) for a single animal. For example, for mammalian cortex we have a good notion on the types of synaptic neurotransmitters/receptors for major classes of cortical neurons based solely on their morphology [12]. However, we do not have a clue how these neurons are individually wired; at best we have large-scale connectivity maps on a level of cortical areas [2]. Contrary, for the nematode *Caenorhabditis elegans* we have a precise neuron-to-neuron connectome [5, 6], but we do not know the type of synaptic signaling between neurons. Moreover, the problem in *C. elegans* is additionally complicated because, unlike in cortical neurons, the same neuron can send different types of neurotransmitters to different postsynaptic targets [13, 14]. In such circumstances, the postsynaptic currents originating from the same presynaptic neuron can be either excitatory or inhibitory, depending on neurophysiological or molecular details of the postsynaptic receptors. Consequently, to acquire the information about the pattern of synaptic signaling in *C. elegans*, one has to consider polarities of connections, instead of the polarities of neurons, which however is much more challenging. This is necessary if we want to understand neuronal basis [15] of low dimensional, stereotyped, and conserved behavior of these worms [16, 17, 18, 19].

The goal of this study is to show that it is possible to determine intrinsic circuit properties based on behavioral characteristics in *C. elegans*, using powerful computational methods. In particular, we want to determine the signs of synaptic connections and input pattern in a small pre-motor interneuron network controlling locomotion. Even in this compact network the space of all possible types of connections (excitation vs. inhibition) is huge. This technical complexity is approached in a reverse engineering fashion, where we compare theoretical computations of neural activities with available behavioral locomotory data, to decipher the synaptic signs. The approach involves the use of optimization technique of genetic algorithms combined with massive parallel computations to increase the automated search speed for finding optimal synaptic patterns that best match the data. This is a novel and an efficient technique, and in principle, it can be applied to other small-scale functional neural circuits in *C. elegans*.

## Results

**Fig 1:**
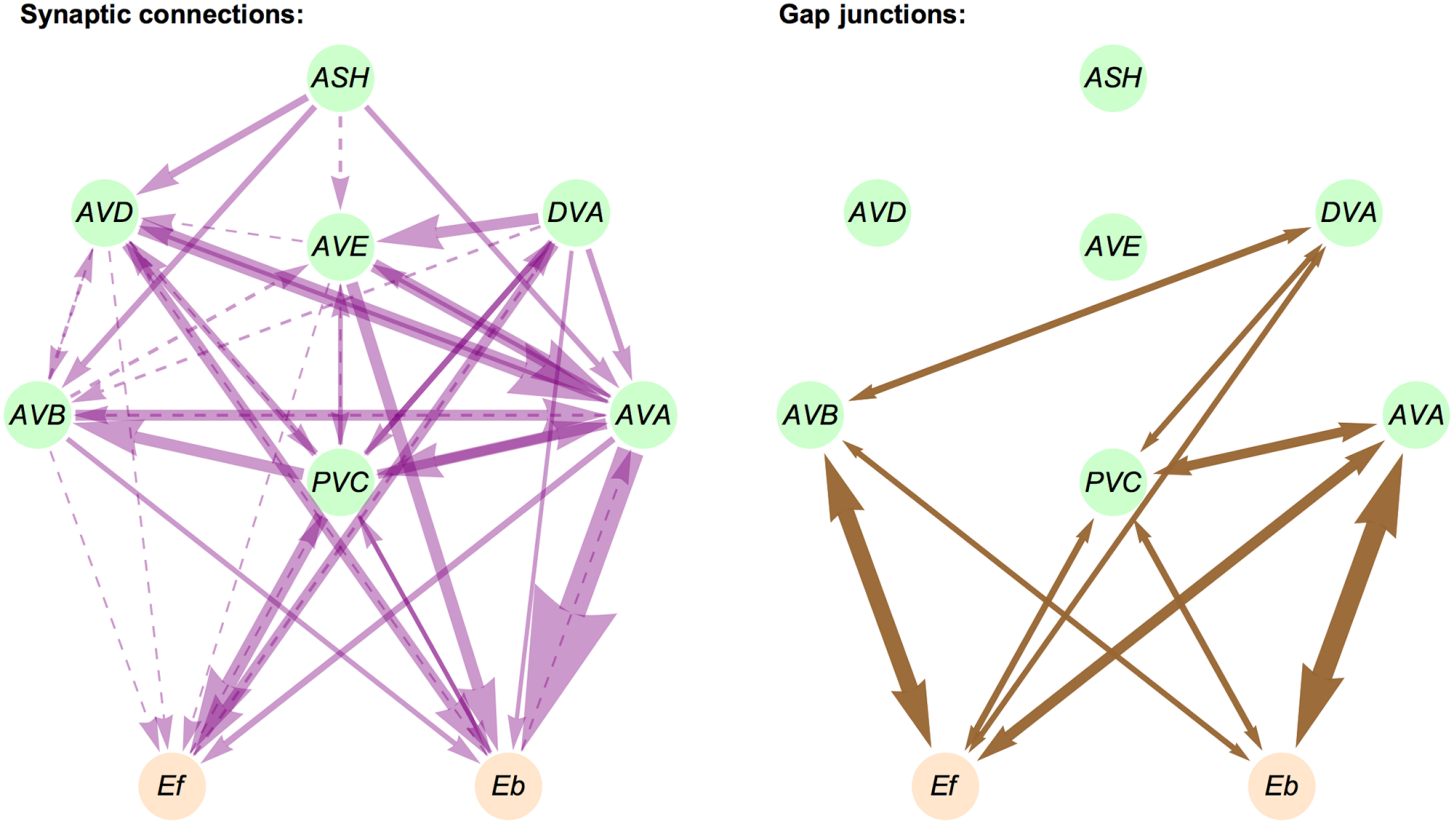
Connectome of a small pre-motor neural network controlling locomotion in *C. elegans*. The strengths of synaptic connections (left panel) and gap junction connections (right panel) are proportional to the thickness of the arrows. Dashed lines for synaptic connections correspond to synaptic strength (average number of contacts) smaller or equal to 0.75.

The primary goal of this study is to determine the most likely synaptic polarities in the locomotor command network of seven *C. elegans* neurons (5 pre-motor interneurons and DVA and ASH) on a connection by connection basis (Fig 1). To achieve this, we computed the relative average times (or relative probabilities) of moving forward and backward from our detailed neurophysiological network model (see the Methods) and compared them with the available experimental times [20]. The comparison was performed for 18 variants of the interneuron circuit: one corresponding to wild type and 17 other versions with reduced number of neurons due to ablations. The theoretical motion times depend on several free physiological parameters, synaptic and gap junction interneuron signaling, and inputs to interneurons. The optimal synaptic polarities are found as a result of minimization of our goal function, which is Standardized Euclidean Distance SED (measuring a generalized distance between theoretical and experimental motion times; see Eq 10 in the Methods) with respect to the free parameters, synaptic signaling, and inputs. The minimization procedure combines Genetic Algorithm method with parallel computations, and it yields a global minimum of the SED function (see Methods).

### The optimal synaptic signaling connectome of the locomotory interneurons

**Fig 2:**
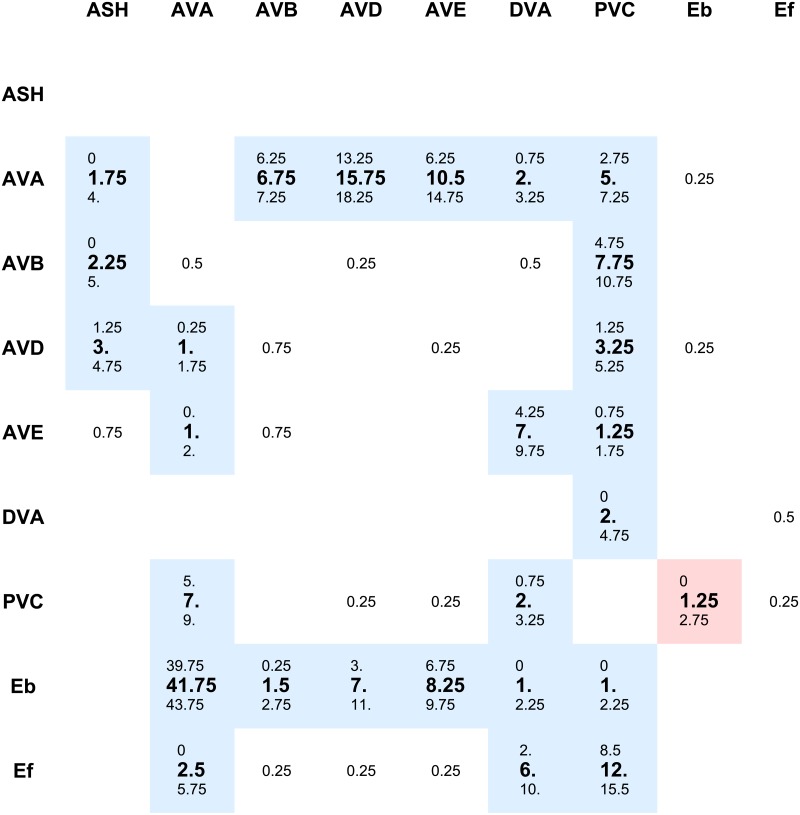
Synaptic connectivity matrix with polarity signs in the pre-motor network. Synaptic strengths between neurons are shown as appropriate matrix elements (columns: presynaptic neurons; rows: postsynaptic neurons). The centered values correspond to mean number of synaptic contacts between neurons, upper values to means decreased by standard deviations, and lower values correspond to means increased by standard deviations. We highlighted in color those synapses whose signs were established by the optimization (the weakest synapses below 0.75 were not optimized). Blue color denotes inhibitory synapses, and red corresponds to excitatory. Note that all optimized connections are inhibitory. The only excitatory connection (E_*b*_ ↦ PVC) is fixed and set by hand, since A and B motor neurons are known to be excitatory [21, 57].

We performed large-scale computations of the command network for two cases: (i) one in which synaptic weights between neurons are fixed and proportional to the mean number of synaptic contacts between them (“mean weights” within two classes of neurons), and (ii) second in which synaptic weights between neurons vary and can take 3 potential values (proportional to the mean number of synaptic contacts, and to mean number either increased or decreased by a standard deviation) from which we pick up one value randomly (“reshuffled weights” within two classes of neurons; see Methods). In both cases, we find that the emerging optimal connectivity signs of the command network are such that all interneuron connections are inhibitory (Fig 2). We were unable to determine the signs of several the weakest connections, due to a combinatorial “explosion” of search time in the huge space of the possible synaptic patterns. However, these neglected weakest synapses do not affect the result in any significant way (see below).

For the case of reshuffled weights, each computation corresponded to a random draw of synaptic weights (see Methods). The detailed results of each computation (with SED values, synaptic polarity patterns, input patterns) are given in Table 1. We find that out of 30 runs only twice we obtain synaptic configurations with mixed polarities (albeit with dominant inhibition). However, judging by very high SED values, these two cases correspond to suboptimal solutions (solutions trapped in local minima). Because the best results for the reshuffled synaptic weights are very close to the result for the case of mean synaptic weights, in the remaining part of the paper we present a detailed neurophysiological analysis only for the case of mean synaptic weights.

**Table 1:** Results for synaptic “reshuffled weights” case.

Run no	SED	Synaptic polarity	Input polarity					
			AVA	AVB	AVD	AVE	DVA	PVC
1	5.570	all inhibitory	-1	1	1	1	1	1
2	5.549	all inhibitory	-1	1	1	1	1	1
3	5.658	all inhibitory	-1	1	1	-1	1	1
4	5.568	all inhibitory	-1	1	1	1	1	1
5	5.470	all inhibitory	-1	1	1	1	1	1
6	5.600	all inhibitory	-1	1	1	1	1	1
7	12.98	all inhibitory	-1	1	1	1	1	1
8	5.698	all inhibitory	-1	1	1	1	1	1
9	5.426	all inhibitory	-1	1	1	1	1	1
10	6.414	all inhibitory	-1	1	1	-1	1	1
11	5.874	all inhibitory	-1	1	1	-1	1	1
12	10.42	most inhibitory*	-1	1	1	1	1	1
13	5.667	all inhibitory	-1	1	1	-1	1	1
14	13.86	all inhibitory	-1	1	1	-1	1	1
15	6.678	all inhibitory	-1	1	1	1	1	-1
16	5.662	all inhibitory	-1	1	1	1	1	1
17	9.033	all inhibitory	-1	1	1	-1	1	-1
18	54.81	most inhibitory**	-1	1	-1	1	1	-1
19	5.672	all inhibitory	-1	1	-1	1	1	1
20	6.564	all inhibitory	-1	1	1	1	1	1
21	6.463	all inhibitory	-1	1	1	1	1	-1
22	12.18	all inhibitory	-1	1	1	-1	1	-1
23	5.296	all inhibitory	-1	1	1	-1	1	1
24	6.658	all inhibitory	-1	1	1	-1	1	-1
25	6.129	all inhibitory	-1	1	1	1	1	1
26	7.033	all inhibitory	-1	1	1	1	1	-1
27	5.641	all inhibitory	-1	1	1	1	1	1
28	5.946	all inhibitory	1	1	-1	1	1	1
29	6.966	all inhibitory	-1	1	1	-1	1	-1
30	6.026	all inhibitory	-1	1	1	1	1	1

* Except excitatory connections: AVA ↦*E*_*b*_, AVA ↦ PVC, AVB ↦*E*_*b*_, AVD ↦ AVA, AVD ↦*E*_*b*_, PVC ↦ AVE, PVC ↦ DVA.

** Except excitatory connections: AVA ↦*E*_*b*_, AVA ↦ PVC, DVA ↦ AVE, PVC ↦ AVB, PVC ↦ AVD.

**Fig 3:**
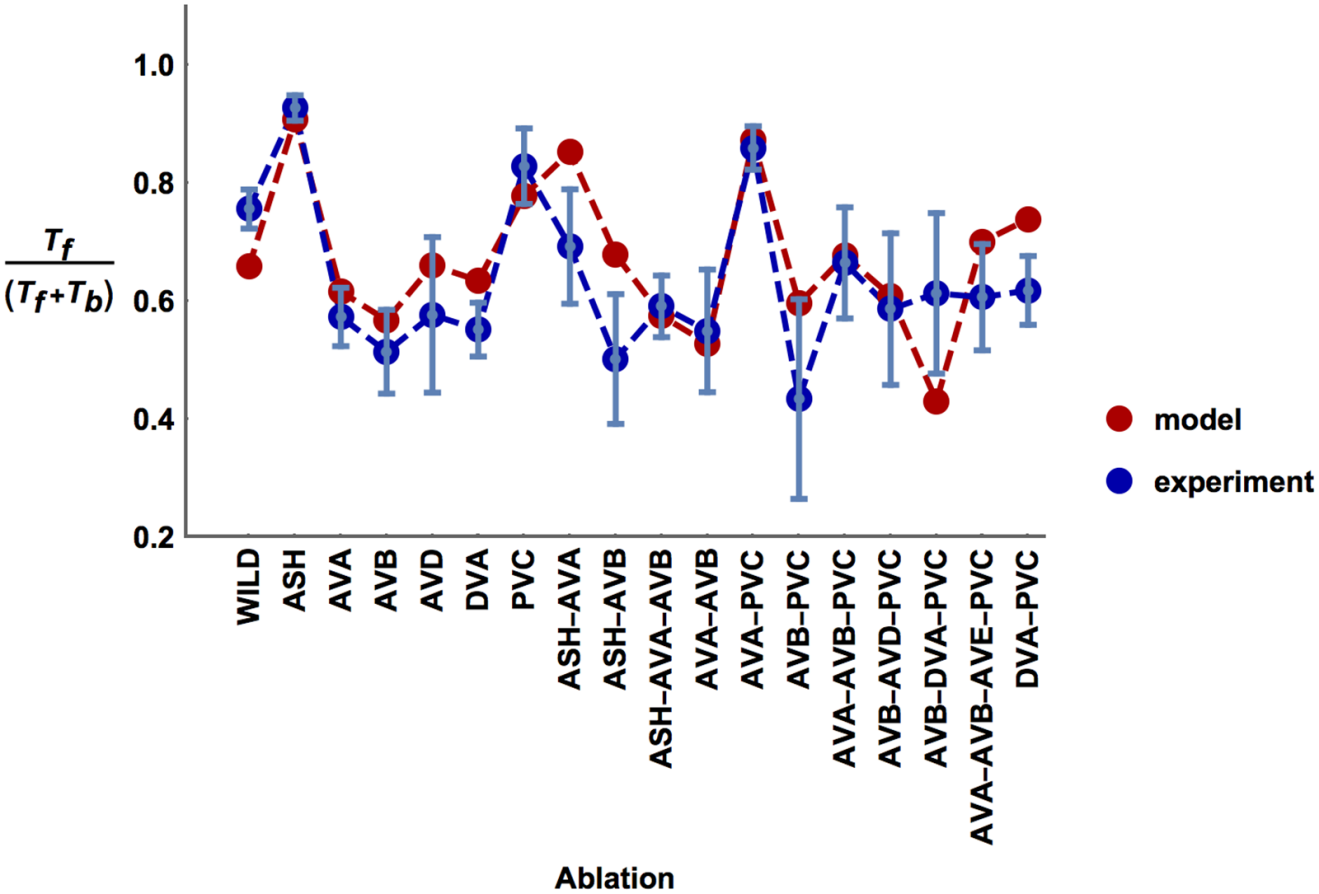
Comparison of computational and experimental ratios of times for moving forward and backward across ablation types. Computational forward vs. backward timings, i.e. *T*_*f*_/(*T*_*f*_ + *T*_*b*_), (red points) correspond to the model with optimized parameters and synaptic polarities. Note that most of the computational points lie within standard deviations of the experimental points (blue).

For the case of mean synaptic weights, the optimal values of the free physiological parameters minimizing SED are given in Table 2. In a related Fig 3, we display a comparison of the experimental ratios of motion times with the optimal theoretical predictions of these ratios for each perturbed version of the circuit. There is a high degree of similarity between these values as most theoretical points lie within standard deviation intervals of the experimental points (Fig 3). In quantitative terms, the optimal connectivity configuration corresponds to the minimal value of SED equal to 5.36. For a comparison, to have a sense of how large this number is, we would obtain SED = 4.24 if distances between theoretical and empirical points were exactly equal to the standard deviations of the empirical values.

**Table 2:** Optimal values of neurophysiological parameters for “mean weights” case.

Variable	Value	Units
*q*_*s*_	0.039	mS/cm^2^
*q*_*e*_	0.042	mS/cm^2^
*X*_*o*_	3.5	*μ*A/cm^2^
*c*_*ash*_	0.5	unitless
*f*_*ash*_	-0.8	unitless
*η*	2.0	mV

**Fig 4:**
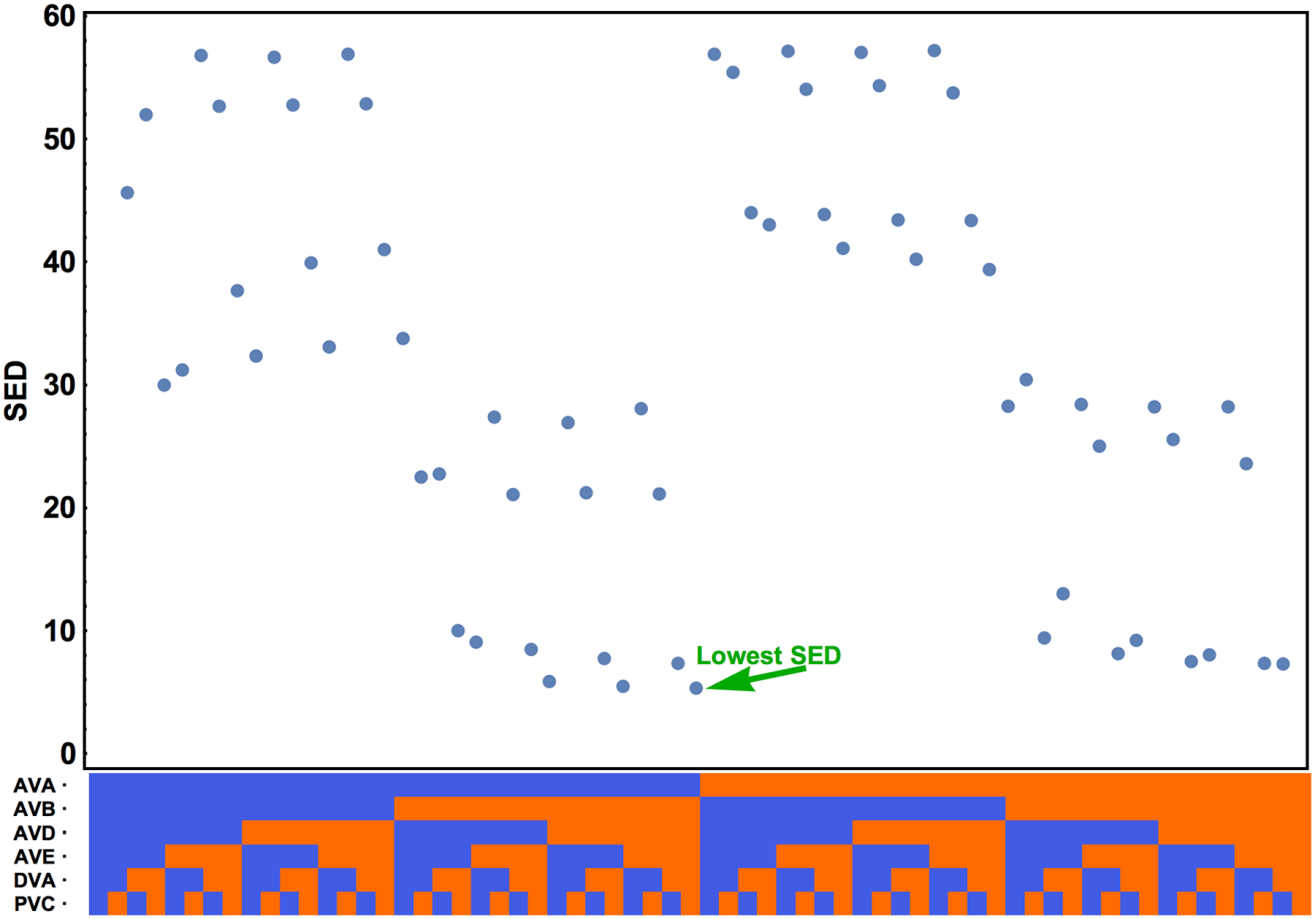
Influence of input pattern on the goal function SED. For the optimal synaptic configuration (all interneuron connections inhibitory) we varied the upstream input pattern (horizontal axis—bottom) and observed the change in SED value. Input to a given interneuron can be either excitatory (in red) or inhibitory (in blue), which gives 64 possible input patterns. The minimal SED is obtained when all interneurons except AVA get excitation.

The optimal input pattern to the interneurons corresponding to the global minimum of SED is shown in Table 3 (mean synaptic weights case). Almost all of the incoming currents are excitatory, except an inhibitory input coming to AVA neuron, which is known as the main driver of backward motion [21]. Interestingly, we obtain the same input pattern for the best solutions in the “reshuffled weights” case (see Table 1). More generally, if we fix the interneurons interconnectivity as inhibitory, the best fits to the behavioral data (the lowest SED values) are achieved for input configurations in which excitation to interneurons dominates (Fig 4). Configurations with prevailing inhibitory input generate the values of SED several-folds higher (Fig 4).

**Table 3:** Optimal input pattern to pre-motor interneurons (*σ*_*i*_) for “mean weights” case.

AVA	-1
AVB	1
AVD	1
AVE	1
DVA	1
PVC	1

### Reduction of synaptic combinatorial space and its influence on the results

The results in Figs 1–3 were obtained by a slight reduction of combinatorial space for synaptic connections in the interneuron circuit (synaptic connection between two interneurons is defined here as a physical link between them with strength proportional to the number of linking synapses). If each synaptic connection is either excitatory or inhibitory, we have 2^38^ ≈ 2.7 ⋅ 10^11^ of possible synaptic patterns of the circuit (there are 38 connections between interneurons). This, however, is too big space for finding the optimal connectivity pattern within a reasonable time even for the fastest available computers. In order to make the problem more tractable, we reduced the combinatorial space by removing from the circuit the weakest connections. Technically, this was implemented by introducing a certain cut-off for connection weight (average number of synaptic contacts), and neglecting those connections with weights equal to or below that cut-off, which was set at 0.75. Because there are 12 such sub-threshold connections, we could reduce the synaptic combinatorial space by the factor 2^12^ = 4096, which enabled us to conduct parallel computations and to obtain the results within an hour of real time.

**Fig 5:**
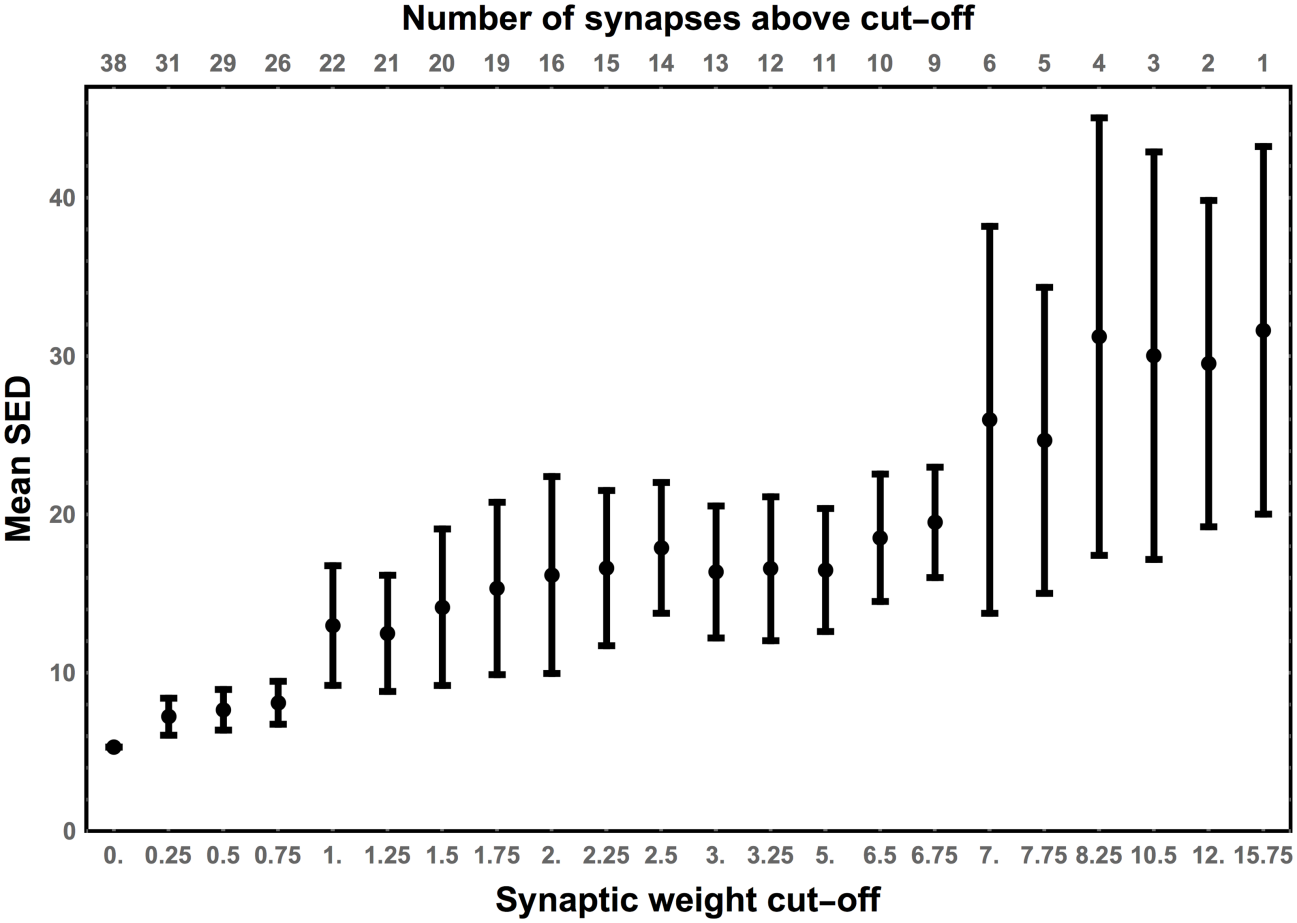
Sensitivity of SED on the synaptic cut-off weight. Each point corresponds to the mean SED for an ensemble of wild type circuits with the following property: all connections whose strengths are above a given cut-off (horizontal axis) are set as inhibitory, while the polarities of the remaining connections are randomly chosen (100 versions). Lowering the synaptic cut-off generally decreases mean SED and its variability. Note that for the cut-off weights ≤ 0.75 the value of SED does not change much.

The rational for choosing the cut-off at 0.75 was based on the dependence of SED value on the number of inhibitory connections and their weight in the circuit (Fig 5), since based on a previous work we anticipated that inhibition would be a dominant synaptic signaling [20]. The goal was to fix the number of inhibitory links above a certain cut-off weight level and vary randomly the polarities of the remaining connections. In a version of the circuit in which the strongest connection is inhibitory and the rest are random (the high cut-off at 15.75), the average SED and its variance are very large (Fig 5). In the opposite case, when all synapses are inhibitory (the smallest cut-off at 0), the SED is the smallest. The general trend is such that decreasing the cut-off (equivalent to increasing the average number of inhibitory connections) causes steady decrease in the average SED and its variance. The SED variability for the cut-offs in the range 0.0−0.75 is relatively constant and substantially smaller than SED for higher cut-offs (Fig 5). This suggests that the cut-off 0.75 is the best in terms of simultaneous computational time and accuracy of the results.

### Sensitivity of the goal function on varying synaptic polarities

**Fig 6:**
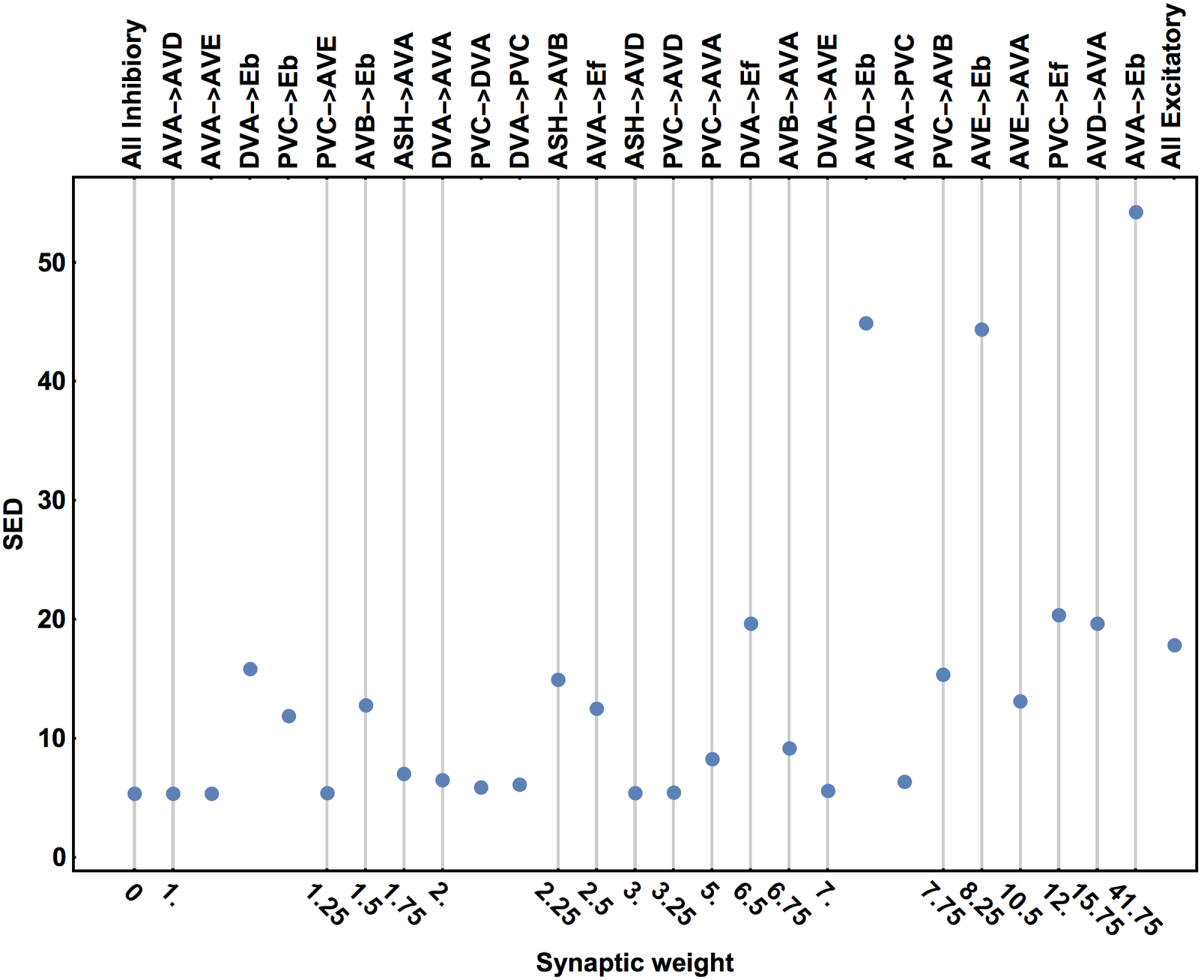
Effect of single synaptic polarity switch on SED. Switching the polarity of a single connection above a baseline with all inhibitory connections can have diverse impact on SED, from vary weak to very strong. The switched connections are labeled in the top and their corresponding strengths are shown in the bottom. The first point on the right corresponds to the configuration with all excitatory synapses. For all points, the inputs to the interneurons are fixed and optimal.

In Fig 6 we show how the goal function SED depends on flipping of the polarity of a single connection, taking as a baseline the optimal configuration with all inhibitory connections (i.e. those with average number of contacts above the threshold 0.75). The polarity switch of the strongest connections (from negative to positive) has a large effect on SED. In particular, reversing the sign of AVA ↦ E_*b*_ synapses causes a huge increase in SED by a factor of ∼ 10. This clearly indicates that this connection cannot be excitatory. Similarly, switching the polarities in the AVD ↦ E_*b*_ and AVE ↦ E_*b*_ connections has a comparative big impact on SED. Interestingly, when all connections in the circuit are excitatory then SED is about 3-fold larger than for the all inhibitory configuration, but it is smaller than for switching the above strong connections terminating at E_*b*_ (A type motor neurons).

Reversing a single polarity for weak and moderate connections can have a mixed influence on the SED value (Fig 6). Switching some of the connections has a little effect, while switching others can substantially affect SED. The largest values of SED for these weak and moderate synapses are on the level of SED for the all excitatory configuration. This implies that there is a chance that some of the weakest connections could be excitatory, e.g. AVA↦ AVD, AVA↦ AVE, and PVC↦ AVE (Fig 6).

### Sensitivity of the goal function on input strength and conductances

**Fig 7:**
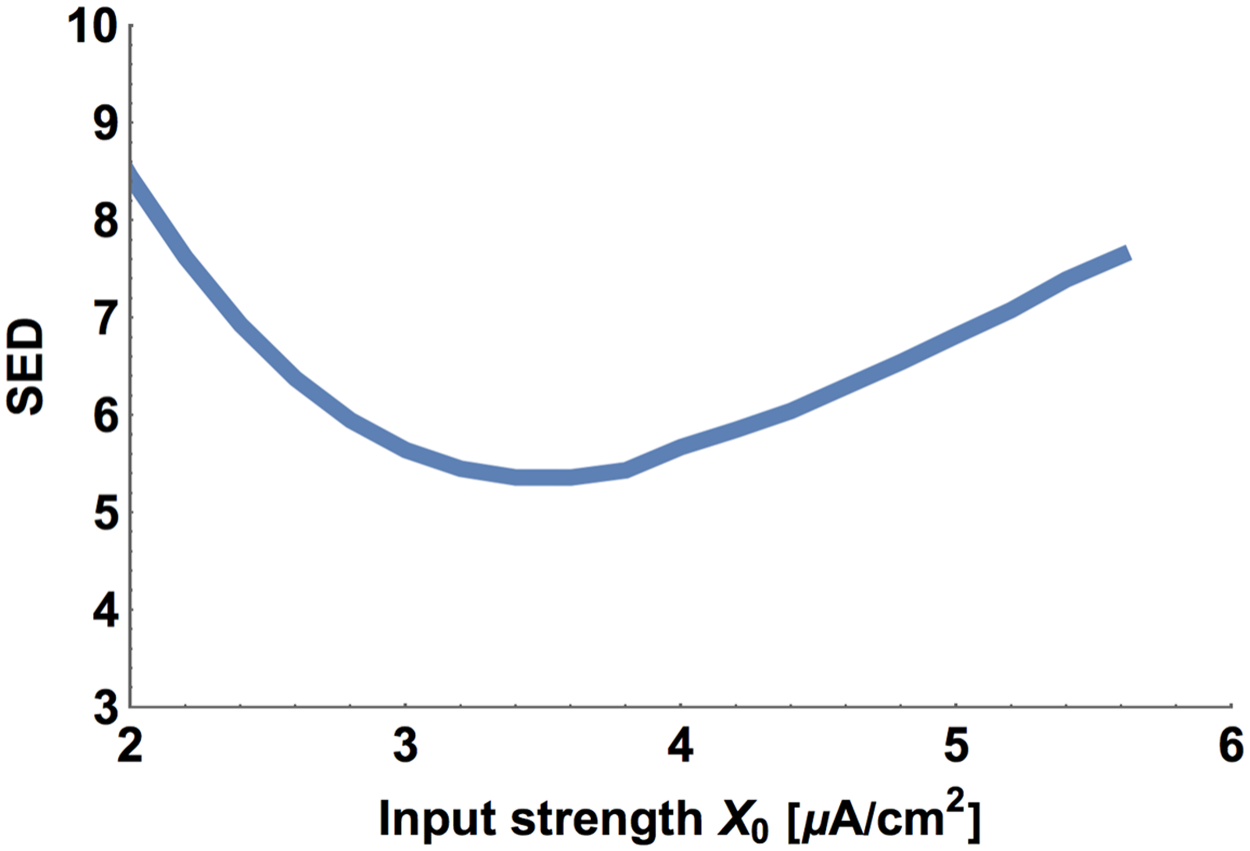
SED as a function of input strength. All other circuit parameters are kept fixed as optimal.

**Fig 8:**
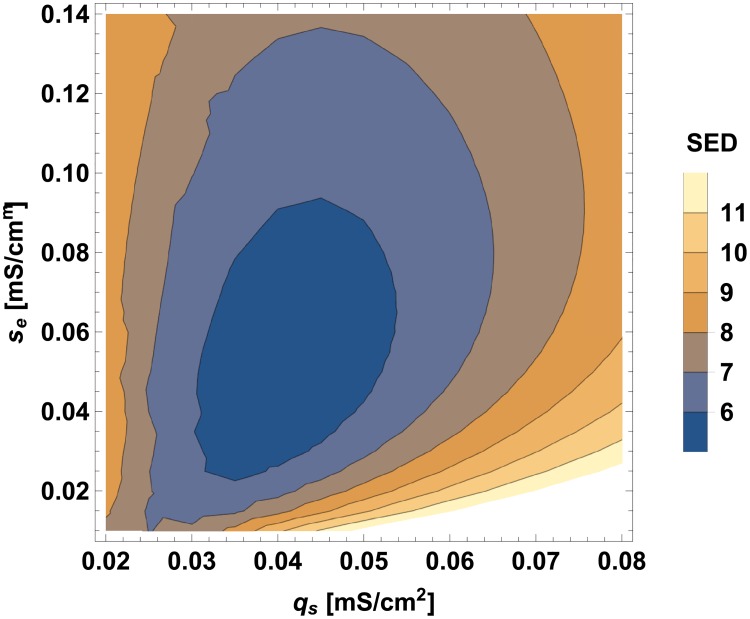
SED as a function of synaptic and gap junction conductances. All other circuit parameters are kept fixed as optimal.

**Fig 9:**
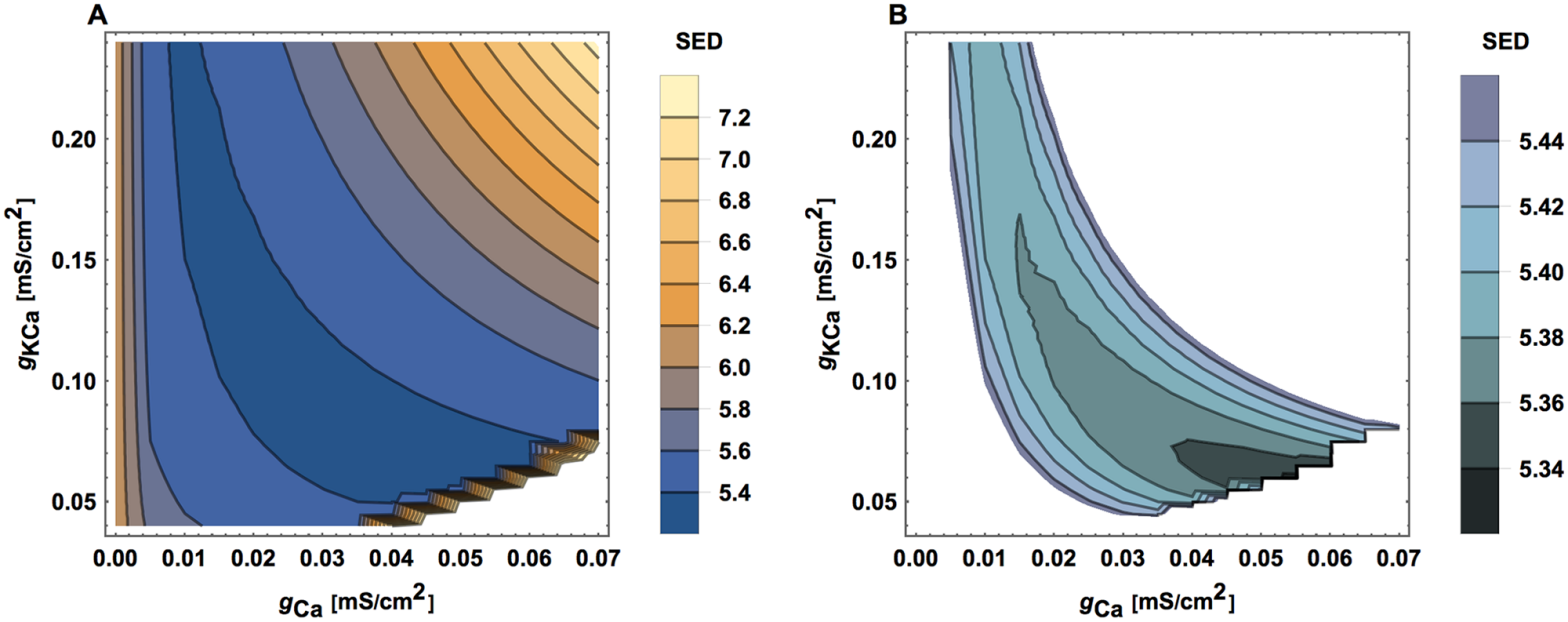
SED as a function of calcium related ionic conductances. Similar as in Fig 8. (A) Global view and (B) more local view.

A similar sensitivity analysis can be performed for the input amplitude (*X*_*o*_), for the conductances associated with connections (*q*_*s*_ and *q*_*e*_), and for the conductances related to calcium (*g*_*Ca*_ and *g*_*KCa*_). For changing values of these parameters (while keeping fixed the optimal values of other parameters), we can observe the changes in the goal function SED (Figs 7–9). A clearly visible minima of SED can be noticed, implying that there are optimal values of these neurophysiological parameters and their optimality ranges are rather narrow.

### The impact of virtual ablations on the distribution of neural activities in the circuit

**Fig 10:**
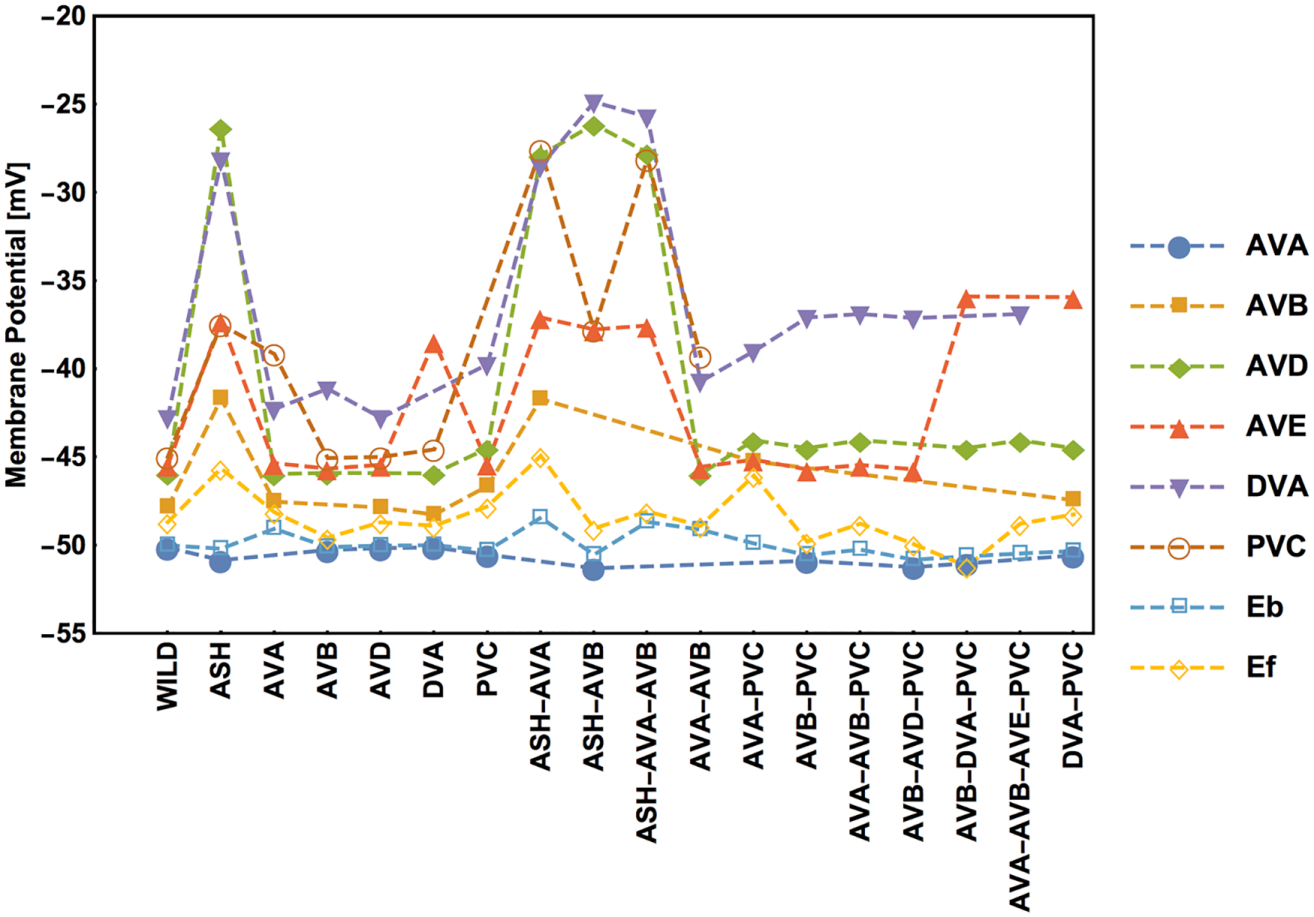
Stationary membrane potential of each interneuron depends on ablation type. Values of membrane potentials are computed for optimal parameters, synaptic polarities, and inputs. The lack of mark for a given ablation denotes the lack of neuron in the reduced circuit.

**Fig 11:**
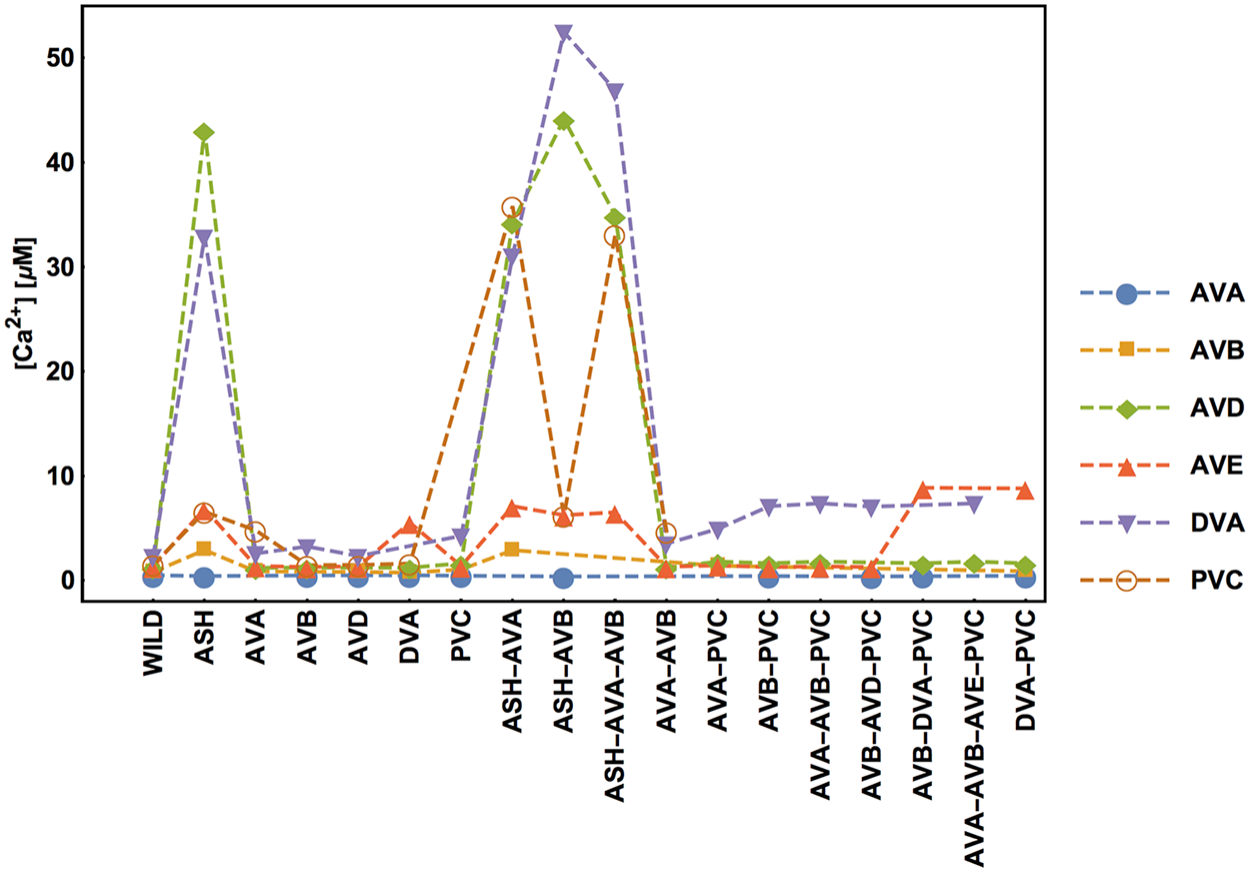
Stationary calcium concentration of each interneuron depends on ablation type. This is analogous to Fig 9.

Every (virtual) removal of neurons and their connections from the circuit causes redistribution of activities of the remaining neurons. The optimal values of stationary membrane potentials and Ca^2+^ concentrations across different ablation types are shown in Figs 10 and 11. There exists a high correlation between neural membrane voltages and intrinsic calcium activities, suggesting that Ca^2+^ concentration can indeed be used as a proxy for neural electric activity. The biggest impact on the optimal activities has the removal of the polysensory neuron ASH, either alone or with other neurons (AVA, AVB). Eliminations of this type cause the largest spreads in the neural activities (Figs 10 and 11). Interestingly, the highest activities across all circuit versions are present in the DVA and AVD neurons, and the lowest in the AVA neuron. The fact that AVB has higher membrane potential than AVA is not intuitive, given that they both inhibit synaptically the downstream motor neurons. However, it should be remembered that AVB inhibitory synapses to motor neurons are extremely weak (Fig 2 and [20]), suggesting that AVB uses instead a gap junction coupling (relatively strong) for that signaling. As a consequence, the average membrane potential *E*_*f*_ of motor neurons B promoting forward motion is always slightly greater (several mV) than the average potential *E*_*b*_ of motor neurons A promoting backward motion (Fig 10). Finally, note that the optimal values of the potentials and Ca^2+^ concentrations are in the ranges expected from neurophysiological studies in other animals [22, 23], which generally suggests that optimized and non-optimized conductances in the model have realistic values.

### Distributions of current flows in the circuit

**Fig 12:**
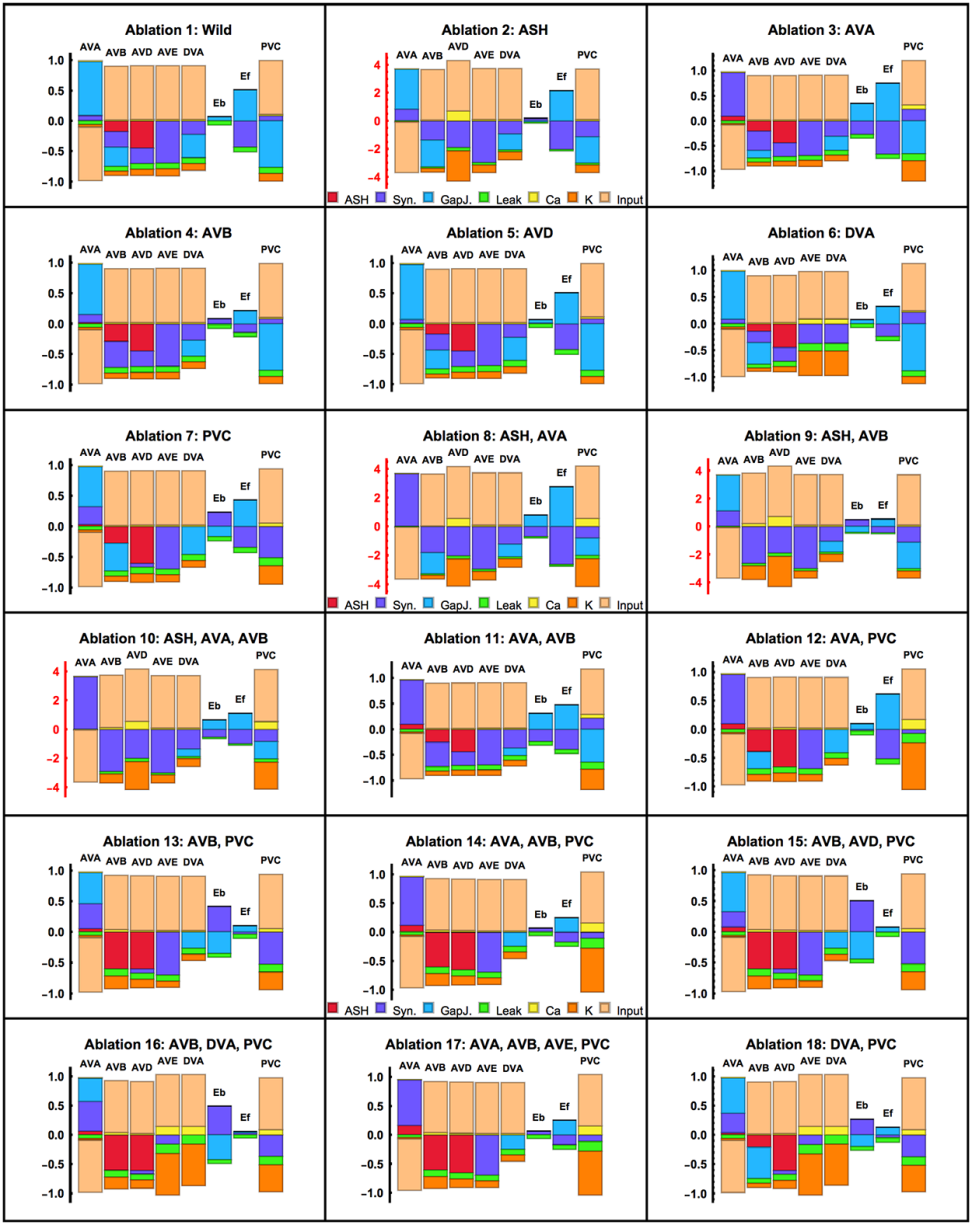
Stationary distributions of currents in neurons across ablations. In a stationary state, all currents passing through a neuron’s membrane must sum up to zero, therefore each bar in the graph has equal positive and negative parts. The currents corresponding to different mechanisms are marked by different colors. Note a different y-axis scale in the subgraphs related to ablations involving ASH neuron.

Related to neural activities are electric currents passing through membranes (Fig 12). For each version of the circuit we can determine stationary values of the synaptic, gap junction, and ionic currents for a given neuron, and compare their relative magnitudes and signs. These current configurations are variable both for pre-motor and for motor neurons. For example, the wild type circuit is dominated by upstream input currents (Fig 12). Moreover, that circuit has the biggest positive synaptic currents flowing into AVA and B motor neurons (*E*_*f*_). In the case of AVA, this synaptic current must be balanced by a large negative input current. Contrary, all other pre-motor neurons receive negative synaptic currents of different magnitudes, which are balanced by large positive inputs. The overall picture is such that the ionic currents (leak, Ca^2+^, and Ca^2+^ activated K^+^) provide only a minor contribution in relation to input and synaptic currents. Gap junctions contribute moderately, which is related to the fact that their numbers are smaller than that of chemical synapses.

**Fig 13:**
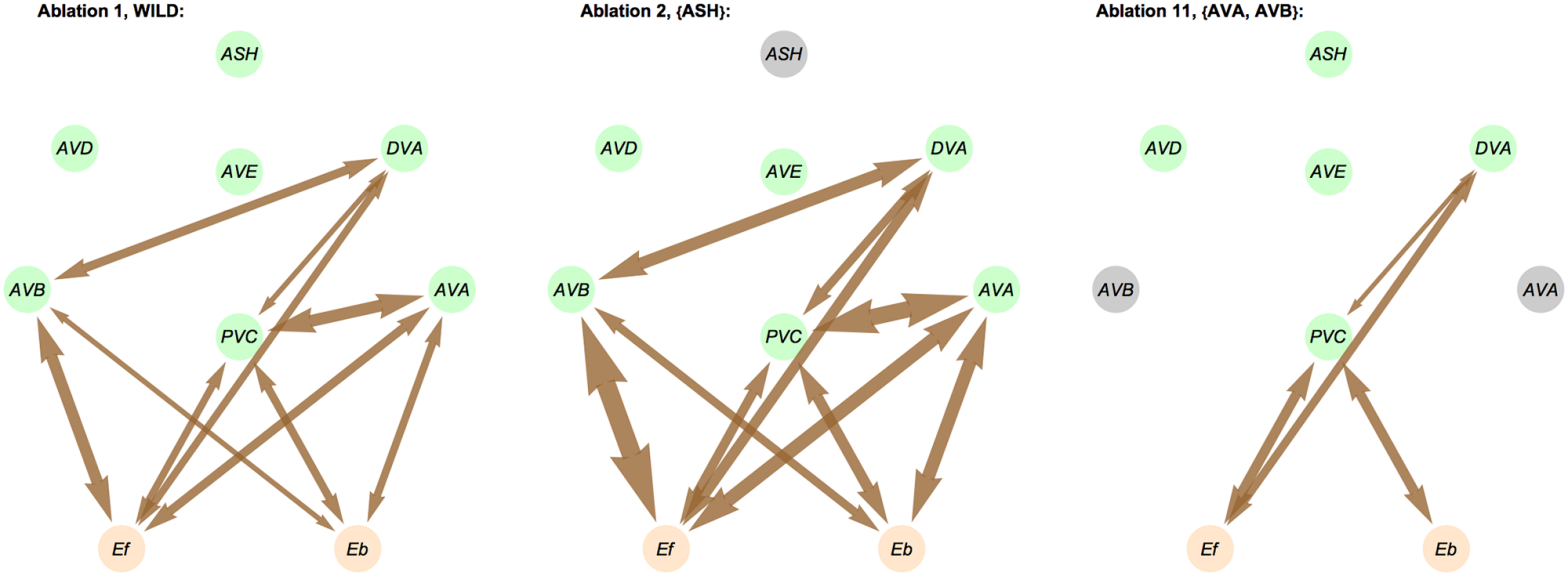
Examples of gap junction currents for wild type and ablated circuits. Arrow width corresponds to the current magnitude. Ablated neurons are colored in black.

**Fig 14:**
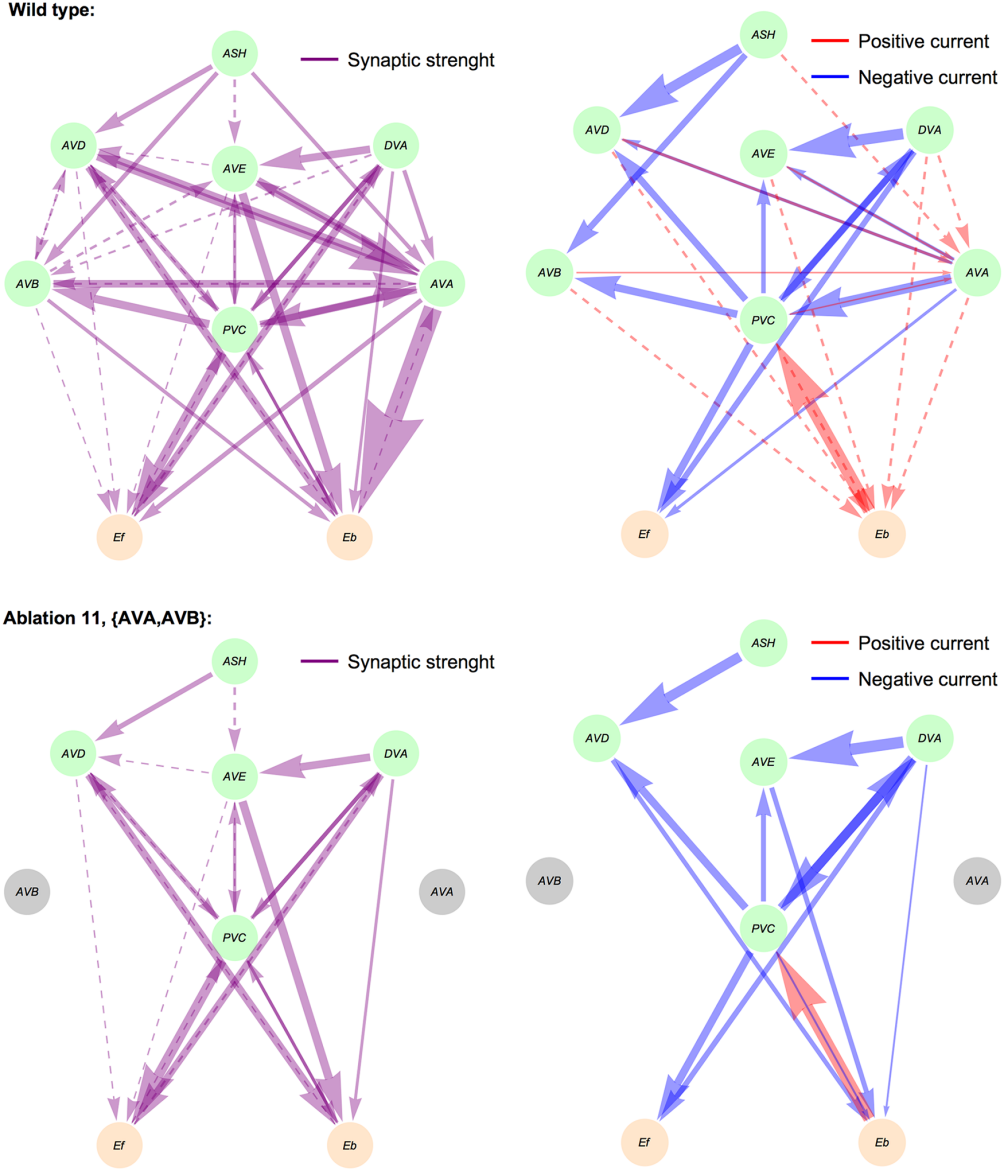
Examples of synaptic currents for wild type and an ablated circuit. For two selected ablation types we show synaptic weights (left column) and synaptic currents (right column) as arrows with widths proportional to their strengths. Weak synaptic connections (below 1.0) and weak synaptic currents (below 0.01 *μ*A/cm^2^) are shown as dashed lines.

Examples of spatial distributions of gap junction and synaptic currents are presented in Figs 13 and 14. For the wild type circuit, the strongest gap junction currents are between AVB and E_*f*_, and between AVA and PVC, which correlates with the strength of these connections, although the strongest gap junction (between AVA and E_*b*_) does not exhibit the strongest current (Fig 13). A similar pattern we obtain for wild type synaptic currents, i.e., the strongest currents are related to the strongest synaptic weights, which are AVA↦E_*b*_ and AVD↦AVA (Fig 14).

## Discussion

### Synaptic inhibition as the optimal signaling in the small locomotory connectome

We have known the *C. elegans* connectome for 3 decades [5], yet it has helped us only moderately in understanding the worm’s locomotory behavior (e.g. [15, 19, 20, 24, 25, 26]). The main reason for this is that the knowledge of the detailed connectivity does not automatically translate itself into the knowledge of neural dynamics. The same circuit with fixed neuroanatomical links can generate diverse patterns of neural activity depending on the type of synaptic signaling, neuromodulation or incoming input, as is evident from experimental and computational studies of small systems (e.g. [9]). Thus, the physical skeleton of neural connections is clearly necessary but not sufficient for reliable modeling of neural functions [9, 10, 11]. Therefore, the determination of the synaptic receptor/neurotransmitter types or synaptic polarities in the connectome is a critical step in the long process of relating neural modeling to animal behavior.

This computational study finds that the optimal pattern of connections between pre-motor interneurons in *C. elegans*, that best fits the data, is exclusively inhibitory. More precisely, we find that every pair of interneurons is connected by inhibitory synapses, provided their link is not too weak. This result is obtained by a kind of reverse engineering, in which we predict the circuit connection signs based on behavioral data. Specifically, we used a combination of theoretical large-scale optimization (Genetic Algorithm and parallel computations) with behavioral locomotory data [20], and we considered different reduced versions of the basic “wild type” circuit. It is important to stress that our findings do not require fine tuning of unknown neurophysiological parameters. On the contrary, the values of the most uncertain parameters are optimized and emerge as a result of the global minimization of the SED function (similarity measure between theory and data; see the Methods), and hence they can be viewed as optimal.

We acknowledge, that there is a certain likelihood that some of the weakest synaptic connections in the pre-motor circuit might be excitatory (e.g. AVA↦AVD, AVA↦AVE, PVC↦AVE, PVC↦DVA, PVC↦AVD, ASH↦AVD). This is possible, since switching their synaptic polarities has a minor effect on the value of the goal function (Fig 6). Nevertheless, even if this were the case, the huge majority of the circuit connections should still be inhibitory.

Our computational result on the dominant inhibitory connectivity between all locomotor interneurons agrees qualitatively with earlier experimental suggestions that pre-motor circuit should use synaptic inhibition as a main mode of interneuronal signaling and act essentially as an inhibitory switch [27, 28]. However, a direct neurophysiological evidence has been lacking. A recent study by Pereira et al [29] shows that the major neurotransmitter used by *C. elegans* neurons, including all its locomotory interneurons, is acetylcholine, which can act both as excitation and inhibition depending on a postsynaptic receptor type. At present there are known several Ach-gated chloride channels (receptors: acc-1 up to acc-4, and lgc-46 up to lgc-49 [30, 31]) that can mediate inhibition (influx of Cl^−^ ions decreases membrane voltage). For example, the receptor acc-1 is expressed in AVA and AVE [29], suggesting inhibitory incoming connections to these neurons, which for AVA agrees also with the optimal polarity of the input it receives (Tables 1 and 3). Interestingly, the connection from AVA to one of A-class motor neurons (AVA ↦ *E*_*b*_ in the model) has been recently found by electrophysiology to be mediated by lgc-46 chloride receptor, and thus the connection is presumably inhibitory [32]. However, more experimental data is needed to confirm our prediction for all pairs of interneurons (including motor neurons).

### Strategy for locomotory decision making in *C. elegans*

The small locomotory interneuron network is a decision making circuit for *C. elegans* motion [33]. These neurons control when the worm moves forward, backward or stops. The discovery of the dominant role of inhibitory synaptic signaling in this circuit along each interneuron connection indicates that decisions to move in a particular direction are made based on restraining the undesired neural activities through blocking of particular links in the network. This mechanism is reminiscent of the “winner takes all” control and it is similar to the decision making in the cerebral cortex of mammals where cross-inhibition plays a key part [34]. Moreover, because of an additional presence of gap junctions in the command circuit, whose main action is to spread activation from excitatory sensory input to motor neurons, the whole locomotor interneuron circuit acts as a push-pull network. This is similar to a push-pull control of motor output in mammalian motor networks [35], and networks of this type exhibit high flexibility in constraining the upstream stimulation (e.g. [36]). In our circuit, the gap junctions provide “push” and inhibitory synapses generate “pull”. This push-pull architecture may be evolutionary advantageous in terms of energy savings, since it is known that neural activities are metabolically expensive [37], and energy cost scales up with network size [38]. Consequently, the suppression of interneuron activities associated with complementary actions not only facilitates decision making but can also substantially save the network energy.

The idea of reciprocal inhibition of the interneurons taking part in opposite directions of motion, advocated here, is consistent with simultaneous calcium imaging from pairs of interneurons in freely moving worms. Specifically, imaging neural activity from AVA and AVB [39, 40], and from AVB and AVE [41], shows that these neurons are anticorrelated. Moreover, the energy efficient winner takes all mechanism suggested here for locomotion control is in line with a recent study on sparse coding of chemosensory information in *C. elegans* [42]. These authors showed that only a small fraction of sensory neurons responds to a given stimulus, which may imply that these neurons interact mostly by inhibition and thus save energy.

### Relation to previous similar computational studies and current computational advancement

The inhibitory nature of the locomotor interneuron network was predicted before based on neurophysiological modeling combined with direct measurement of locomotory characteristics [20]. A similar suggestion was made for *C. elegans* tap withdrawal circuit using a distinct computational method but a similar spirit of the reverse engineering approach (deciphering intrinsic network properties using behavioral characteristics) [43]. The current study further validates and extends the previous result in [20] in five important ways.

First, experimental studies in *C. elegans* show that many of its neurons can produce diverse types of neurotransmitters and receptors simultaneously, and hence there is a possibility that the same neuron can excite some of its postsynaptic partners and inhibit other [13, 31]. Therefore, we consider the polarity of each connection between interneurons instead of the polarity of each interneuron as was done in Rakowski et al [20]. From a theoretical point of view, this is an important conceptual advancement because up to now such a possibility was virtually neglected in the long tradition of modeling in theoretical neuroscience [44]. This seems to be necessary for future more realistic modeling of neural dynamics in *C. elegans*.

Second, the shift from neuron polarity to connection polarity introduces a big computational challenge, since now the combinatorial space of all possible connectivity patterns is dramatically larger (2^38^ vs. 2^7^ in [20]). In the previous study it was enough to analyze the results, i.e. to find minima of a goal function, by “eye inspection” and brute force, without any involvement of sophisticated optimization methods. In the present case, such an approach is infeasible, and to deal effectively with a huge combinatorial space we implemented multidimensional optimization technique based on genetic algorithms and conducted computations in a parallel fashion (PCJ library) to speed them up. This novel and fast computational technique is in our view a major technical advancement that can potentially be used also for analysis of other small functional networks in *C. elegans* (see also below).

Third, the current model of neural dynamics is more complex than the one used in [20]. The main difference is that the previous model was only weakly nonlinear (nonlinearity only in synaptic transmission), whereas the present model is highly nonlinear due to inclusion of calcium and calcium-activated potassium channels. The nonlinear Ca^2+^ effects could easily overwrite synaptic effects, which suggests that the dynamics of neurons in the present model and those in [20] are generally different. Generally, inclusion of calcium makes the model more realistic, since calcium and calcium activated potassium currents have been reported experimentally for *C. elegans* neurons [45], and calcium levels can be related to behavioral decisions of the worm [46]. Moreover, adding calcium dynamics is important in the light of recent experimental progress in using calcium imaging as a tool for monitoring large-scale neural activity of freely behaving worms [47, 48, 49, 50], and it can provide prospects for linking such experiments with detailed neurophysiological modeling.

Fourth, the current study includes some variability in the synaptic weights between neurons due to pairing of interneurons within the same class. This effect was not considered in [20].

Fifth, in this study we use a slightly more general and more accurate measure of the similarity between theoretical predictions and empirical data. It is based on a standardized Euclidean distance SED, which is a particular version of a more general Mahalanobis distance, and it directly takes into account the errors in the data points (see the Methods). The latter feature was absent in Ref. [20].

The computational methodology presented in this paper (relatively fast computations for different versions of the same neural circuit) works well and efficiently if the network size is sufficiently small. What matters for the efficiency is not so much the number of neurons in the network, but the number of connections between them. Our present computational setup can handle up to ∼ 30 connections so that an analysis for a single run can be performed within a reasonable time of ∼ 3 days. The approach would be infeasible for networks with number of connections well above 30. Fortunately, for *C. elegans*, its major functional networks, including chemotaxis [51, 52] and thermotaxis [53] circuits are relatively small, with connection number below or about 30, most of them with unknown polarities. This suggests that the methodology presented in this paper could in principle be used (possibly with some modifications) to decipher synaptic signaling between neurons in these circuits, especially that there are available some calcium imaging data combined with behavioral data [52]. This would require to define a meaningful “goal functions” that could relate empirical data to theoretical predictions, which however should be doable.

In a certain modeling aspect related to locomotory transitions, the current approach is mathematically similar to a recent approach in [54] and the one used in Ref. [20]. In fact, all three papers use the same methodology based on Markov chains (Master Equations) and a small set of behavioral states, using a sort of Arrhenius law which is common in statistical physics [55] (see the Methods). The differences between these approaches are in details, e.g., we focus on stationary solutions corresponding to long-term average behavior, while in Ref. [54] the emphasis is on a short-term non-stationary switching between the states.

### Testable prediction of the circuit model and future prospects for realistic modeling of *C. elegans* behavior

Our neurophysiological model of the interneuron circuit can be used to determine the stationary values of calcium concentrations. We can find these concentrations across different artificial neural ablations, and thus there is a possibility to compare theoretical calcium activities with those measured in imaging experiments. It seems that the most productive comparison would be for the circuits with ablated ASH neuron (possibly with other neurons), since for these perturbations we predict a wide separation in Ca^2+^ levels: high activities for the DVA and AVD neurons, and low activity for the AVA neuron, with AVB calcium level somewhere in between.

In contrast to the view expressed in Ref. [54] that there is no enough neurophysiological data to model *C. elegans* behavior on a single neuron level, we do believe that it is feasible as the current study shows. The lack of certain neurophysiological data can be circumvented by an optimization technique, such as the one presented here, which would enable us to “fix” certain critical parameters in such a way that the output of a given circuit agrees with some behavioral observation. This is precisely what we do in this study; we optimize the circuit performance in order to match it to the locomotory data. A similar strategy can be applied to other functional circuits in *C. elegans*, as it was applied to its klinotaxis system [19]. We also hope that the determination of the synaptic signs in the pre-motor circuit can be used for improving or extending modeling studies related to neural control of locomotion. Specifically, the issue of the relative contributions of a Central Pattern Generator (CPG) and sensory feedback to creating body oscillations could be addressed anew (see e.g. [25, 26, 56]).

## Methods

The ethics statement does not apply to this study, since we do not perform any experiments.

### The outline of the procedures used

We consider 18 structural versions of the interneuron locomotory circuit, which sends signals to two groups of downstream motor neurons promoting either forward or backward motion. The primary version of the interneuron circuit corresponds to wild type, and the rest are perturbed versions of the circuit with reduced number of its neurons (virtual ablations as in [20]). For each version of the circuit we compute stationary activities of interneurons and motor neurons. The activities of motor neurons are then used as a link between neurophysiological activity and locomotory behavior. We consider 3 locomotory states (forward and backward motions, and stop phase) and for each we compute a corresponding stationary (long-term) probability, using Master equation approach. These probabilities are related to the average times of dwelling in a particular state, which enables us to compare theoretical ratios of dwelling times to available experimental data from [20]. We introduce a similarity measure between theoretical and experimental timings based on a standardized Euclidean distance SED, which serves as our goal function. This function depends on many physiological parameters, as well as on the pattern of connectivity signs, and on the input to the circuit. Using genetic algorithm and parallel computations we minimize the goal function with respect to these three classes of parameters and find their optimal configurations that give a global minimum of SED. As the result of this procedure we find the optimal pattern of the connectivity signs in the circuit, the optimal input pattern, and the optimal values of neurophysiological parameters whose empirical values are not known.

### Model of connectome and determination of synaptic strengths

The network or connectome controlling locomotion in *C. elegans* consists of 5 classes of pre-motor interneurons (AVA, AVB, AVD, AVE, PVC), in each class there are two neurons and we treat them as one “averaged”. Additionally, we consider one sensory dorsorectal neuron DVA, and two polysensory amphid neurons ASH, which we also treat as one (Fig 1). Synaptic weights between classes of neurons (called throughout the paper simply neurons) are determined in the following way. For a given presynaptic (*Y*_*pre*_) and postsynaptic (*Y*_*post*_) class of neurons there are 4 (2 in the case of DVA) numbers of synaptic contacts between them [6]. We compute the mean (*m*) and standard deviation (*sd*) of these numbers, and assume that a synaptic weight between *Y*_*pre*_ and *Y*_*post*_ can take 3 potential values that are proportional to *m*, *m* − *sd*, and *m* + *sd* (Fig 2). This effect takes into account some variability in synaptic contacts between two classes of neurons. Computations for which synaptic weights are proportional only to *m* are called the case of “mean weights”. Computations in which we pick up randomly one of the values *m*, *m* − *sd*, *m* + *sd* for a synaptic weight are called “reshuffled weights”. Most analysis in the paper is performed for the case of mean weights.

### Model of neural activities

The locomotory interneurons signal to downstream motor neurons of type A and B, which are responsible for generating respectively backward and forward motion of the worm [21, 57]. The activities of AVB and PVC interneurons have been traditionally associated with forward motion control, and the activities of AVA, AVD, AVE with backward control [21]. However, now it seems that the issue of directional control is more subtle, because the simultaneous removal of AVB and PVC (by laser ablation), albeit slows down the forward motion significantly, does not abolish it completely [20], suggesting some level of collective control.

We assume that the activities of all 5 command interneurons and sensory neuron DVA are of graded type, since their membranes lack voltage-activated sodium channels [58] and hence they likely lack Na^+^ spikes. These neurons, however, posses other active currents [59], of which those related to Ca^2+^ and K^+^ seem to be the most relevant [45]. The neural activities are represented as:

$$\begin{array}{c} C\frac{dV_{i}}{dt}=-g_{L}\left(V_{i}-V_{L}\right)-g_{Ca}m_{i}^{2}\left(V_{i}-V_{Ca}\right)\\ -g_{KCa}\frac{{\left[Ca\right]}_{i}}{\left(K_{D}+{\left[Ca\right]}_{i}\right)}\left(V_{i}-V_{K}\right)-\sum_{j}\;a_{i}a_{j}g_{ij}\left(V_{i}-V_{j}\right)\\ -\;\sum_{j}\;a_{j}w_{ij}H_{i}\left(V_{j}\right)\left[V_{i}-\left(1-\epsilon_{ij}\right)V_{Cl}\right]+X_{i}\left(V_{ash}\right), \end{array}$$ (1)

where *V*_*i*_ is the membrane potential of neuron *i* and *C* is the membrane capacitance. The first term on the right describes the leak current with conductance *g*_*L*_ and reversal potential *V*_*L*_. The second term is the calcium current with conductance *g*_*Ca*_, reversal potential *V*_*Ca*_, and the gating variable *m*_*i*_ = (1 + exp[−(*V*_*i*_ + 20)/9])^−1^ [60]. The third term is Ca^2+^ activated potassium current with the conductance *g*_*KCa*_, reversal potential *V*_*K*_, calcium concentration [*Ca*]_*i*_, and concentration threshold *K*_*D*_ [60]. The fourth term describes gap junction coupling between neurons *i* and *j* with conductance $g_{ij}=N_{ij}^{e}q_{e}$, where $N_{ij}^{e}$ is the average number of gap junction contacts between *i* and *j* (see [20]) and *q*_*e*_ is the single gap junction conductance per membrane area (it is optimized). The parameter *a*_*i*_ denotes the presence or absence (ablation index) of neuron *i*, i.e., if *a*_*i*_ = 0 then the *i* neuron is removed from the network, and if *a*_*i*_ = 1 then it is present. The fifth term represents the synaptic transmission from neuron *j* to neuron *i* with synaptic strength $w_{ij}=N_{ij}^{s}q_{s}$, where *q*_*s*_ is the synaptic conductance per area (it is optimized), and $N_{ij}^{s}$ is the number of synaptic contacts between *j* and *i*. *N*_*ij*_ can take 3 potential values: mean of synaptic contacts, mean increased by standard deviation of synaptic contacts, or mean decreased by the standard deviation (as described above). The number of synaptic contacts are given explicitly in Fig 2. The function **H_i_**(*V*) is the sigmoid function describing synaptic transmission, and **H_i_**(*V*) = (1 + exp [−*γ_i_*(*V* − *θ_i_*)])^−1^, where *θ*_*i*_ is the threshold for synaptic activation and *γ*_*i*_ is a measure of steepness of the activation slope [22]. The parameters *ϵ*_*ij*_ are elements of the connection polarity matrix (see below), and they can assume values either 1 or 0, depending on the type of synaptic transmission from *j* to *i*. Specifically, if *ϵ*_*ij*_ = 1 then the synaptic connection from *j* to *i* is excitatory, and if *ϵ*_*ij*_ = 0 then the connection is inhibitory. In the former case the reversal potential for synapses is 0, while in the latter it is equal to *V*_*Cl*_ (Cl ions reversal potential because these ions mediate inhibition), which is in agreement with a known neurophysiology [22, 23]. The last term on the right, i.e., *X*_*i*_(*V*_*ash*_) denotes the input current coming to neuron *i* and it is to some extent dependent on the activity level of ASH neuron (see below).

Average activities of motor neurons promoting forward (*E*_*f*_) and backward (*E*_*b*_) motion are given by:

$$\begin{array}{c} C\frac{dE_{i}}{dt}=-g_{L}\left(E_{i}-V_{L}\right)-\sum_{j}\;a_{j}g_{ij}\left(E_{i}-V_{j}\right)\\ -\;\sum_{j}\;a_{j}w_{ij}H_{i}\left(V_{j}\right)\left[E_{i}-\left(1-\epsilon_{ij}\right)V_{Cl}\right], \end{array}$$ (2)

where *E*_*i*_ is the average membrane potential of motor neurons, either *E*_*f*_ (B neurons) or *E*_*b*_ (A neurons). We assume that the A and B motor neurons send out exclusively excitatory synapses, in agreement with experimental suggestions [21, 57].

Activities of calcium concentration in the command interneurons and DVA are given by [60, 61]:

$$\begin{array}{c} \frac{d{\left[Ca\right]}_{i}}{dt}=-\frac{{\left[Ca\right]}_{i}}{\tau_{Ca}}-\frac{2g_{Ca}m_{i}^{2}}{dF}\left(V_{i}-V_{Ca}\right) \end{array}$$ (3)

where *τ*_*Ca*_ is the calcium decay time constant, *d* is the average dendrite diameter of the neurons, and *F* is the Faraday constant.

In total we have 14 differential equations describing the neural and calcium dynamics of 6 pre-motor neurons (except ASH) and average neural dynamics of two groups of motor neurons. These equations are extensions of the models used in [20, 25] and are solved using a second-order Runge-Kutta method. We look for stationary (long-term) solution of this system for different configurations of synaptic polarities and inputs in the pre-motor circuit, and for different versions of the circuit (wild type and reduced). The stationary values of *E*_*f*_ and *E*_*b*_ mediate the neural activity of the circuit to motor output, and they link circuit neurophysiology with behavior (see below).

### Activity of ASH neuron and input to the pre-motor circuit

The ASH neuron is a polysensory amphid neuron located in the anterior part of the worm, sensitive to different environmental factors like nose touch or volatile chemicals. In the experiments from which we have collected data the environment was chemically homogeneous and the worm was not a subject of any mechanical stimulation [20]. Thus, in our computational model we assume that the voltage activity of this neuron *V*_*ash*_ is time independent. The actual value of its activity is set up in relation to the threshold parameter *θ*_*ash*_ of synaptic transmission. We use a simple relation *V*_*ash*_ = *c*_*ash*_*θ*_*ash*_, where *c*_*ash*_ is an additional unitless parameter describing the level of stimulation of the interneuron circuit by the ASH neuron, and its value is optimized. Moreover, ASH vastly projects to many nerve cells in the whole worm, including many head neurons, which in turn project to our circuit interneurons as input *X*_*i*_. As a result, the ASH neuron can influence the downstream interneuron circuit in two distinct ways: either directly by synaptic and gap-junction connections or indirectly through the input *X*_*i*_. Consequently, we express the input as

$$\begin{array}{c} X_{i}=X_{o}\sigma_{i}[1+a_{ash}f_{ash}H_{ash}\left(V_{ash}\right)], \end{array}$$ (4)

where *X*_*o*_ is the input amplitude, *σ*_*i*_ is the vector of input polarities (either 1 or -1), *f*_*ash*_ is the coupling unitless parameter (all three of these parameters are optimized), and *a*_*ash*_ is the ablation index.

### The link between neural activities and behavioral states

Behavioral states of the worm in our model correspond to its 3 locomotory states: movement forward, backward, and stop period. (For simplicity and mathematical clarity, we consider only one stop state, although there are some suggestions that there might be two distinct stop states [17, 54].) Since the worm’s behavior is to a large extent stochastic, we associate with each locomotory state a probability of being in that state. Transitions between the states, or behavioral dynamics, are modeled using a concept of Markov chains, which is a standard tool for describing stochastic phenomena in physical and engineering sciences [55]. In this type of model, the neural activities must be somehow linked to the transition rates between the states. There were some experimental indications [41, 62, 63] that the likelihood to move forward or backward depends on a relative difference in electric activities of the corresponding motor neurons (A and B types [21]). This was also supported by direct measurements and observation of up and down states in voltage membrane of the motor neurons [64]. Specifically, if the activity of B motor neurons is greater than the activity of A neurons, then the worm most likely moves forward. We use these empirical suggestions, and assume that all the transition rates in the model depend on the difference of *E*_*f*_ and *E*_*b*_, i.e., activities of motor neurons promoting respectively forward and backward motions. Moreover, we assume, in agreement with empirical observations, that there are no direct transitions from forward to backward states (and reverse). Transitions of this type are possible only by passing through a stop state, and hence are indirect.

Our model of behavioral dynamics takes the form:

$$\begin{array}{c} \frac{dP_{f}}{dt}=W_{fs}P_{s}-W_{sf}P_{f}\\ \frac{dP_{s}}{dt}=W_{sf}P_{f}+W_{sb}P_{b}-\left(W_{fs}+W_{bs}\right)P_{s}\\ \frac{dP_{b}}{dt}=W_{bs}P_{s}-W_{sb}P_{b} \end{array}$$ (5)

where *P*_*f*_, *P*_*b*_, *P*_*s*_ are instantaneous probabilities of moving forward, backward, and stopping, and they sum up to unity. The coefficient *W*_*αβ*_ denotes the transition rate from the state *β* to the state *α*. Guided by thermodynamic concepts of Arrhenius type transitions [55] and simplicity, we assume the exponential forms of these rates because the exponential function is monotonic and it leads to the sigmoidal shape for an input-output relationship (see below). The transition rates are given by:

$$\begin{array}{c} W_{fs}=W_{o}\exp\left[\left(E_{f}-E_{b}-\Delta \right)/\left(4\eta \right)\right]\\ W_{bs}=W_{o}\exp\left[\left(E_{b}-E_{f}-\Delta \right)/\left(4\eta \right)\right]\\ W_{sf}=W_{o}\exp\left[-\left(E_{f}-E_{b}-\Delta \right)/\left(4\eta \right)\right]\\ W_{sb}=W_{o}\exp\left[-\left(E_{b}-E_{f}-\Delta \right)/\left(4\eta \right)\right], \end{array}$$ (6)

where *W*_*o*_ is a basal transition rate setting the time scale for transitions (in Hz), Δ is some unknown threshold activity (positive) that separates the motion states from the stop state, *η* is the noise amplitude in the system (in mV) and it is a subject of optimization. The choice made in Eq (6) is equivalent to assuming that the worm will likely move forward if *E*_*f*_ − *E*_*b*_ > Δ, move backward if *E*_*b*_ − *E*_*f*_ > Δ, and stop if |*E*_*f*_ − *E*_*b*_| < Δ.

We are interested in the long-term average behavior of the worm, and hence we look for a stationary solution of Eq (5). In this long time limit, ${\dot{P}}_{f}={\dot{P}}_{b}={\dot{P}}_{s}=0$, and we can find an explicit dependence of the average probabilities on the difference *E*_*f*_ − *E*_*b*_. They read

$$\begin{array}{c} P_{f}=Z^{-1}\exp\left[\left(E_{f}-E_{b}\right)/\left(2\eta \right)\right]\\ P_{b}=Z^{-1}\exp\left[-\left(E_{f}-E_{b}\right)/\left(2\eta \right)\right]\\ P_{s}=Z^{-1}\exp\left[\Delta /\left(2\eta \right)\right], \end{array}$$ (7)

where *Z* is given by

$$\begin{array}{c} Z=2\cosh\left[\left(E_{f}-E_{b}\right)/\left(2\eta \right)\right]+\exp\left[\Delta /\left(2\eta \right)\right]. \end{array}$$ (8)

Note that when (*E*_*f*_ − *E*_*b*_)/*η* ≫ Δ/*η*, then *P*_*f*_ ↦ 1 and hence there is a very high probability that the worm moves forward, which is consistent with experiments [41, 62, 63, 64].

Average (long term) times of dwelling in forward, backward, or stopped states, denoted respectively by *T*_*f*_, *T*_*b*_, and *T*_*s*_ are proportional to the corresponding stationary probabilities *P*_*f*_, *P*_*b*_, and *P*_*s*_, since in general we have *P*_*f*_ = *T*_*f*_/(*T*_*f*_ + *T*_*b*_ + *T*_*s*_), etc. These relations imply that the ratio *R* of forward to backward times is given by:

$$\begin{array}{c} R\equiv \frac{T_{f}}{T_{f}+T_{b}}=\frac{1}{1+\exp[(E_{b}-E_{f})/\eta ]}. \end{array}$$ (9)

It is useful to think of the *R* function as the input-output relationship, with *E*_*f*_ − *E*_*b*_ being an activity input, and the ratio *T*_*f*_/(*T*_*f*_ + *T*_*b*_) being a locomotory output. This relationship has a sigmoidal shape (or S-shape), which is typical for many activation processes in biology. Note that 0 ≤ *R* ≤ 1, and *R* ↦ 1 if the time of moving forward is much longer than the time of moving backward. More importantly, the ratio *R* is independent of the unknown threshold Δ. Consequently, we can directly relate the output of our pre-motor circuit to the ratio of average behavioral times the worm spends in forward and backward motions. Different versions of the interneuron circuit with reduced number of neurons cause in general different motor neuron activities, which results in different behavioral dwelling times. This causality can be used to decipher the pattern of synaptic signaling in the circuit. Finally, the same result for *R* was derived in [20], but using a different argument, not invoking Master equation dynamics.

### The goal function

Our goal function relates the theoretical input-output *R* values computed from Eq (9) to the empirical measurements of *R* given in [20]. Specifically, we choose the goal function as the standardized Euclidean distance (a variant of Mahalanobis distance), abbreviated as SED, and represented as

$$\begin{array}{c} \text{SED}=\sqrt{\sum_{i=1}^{18}\;{\left(\frac{R_{th}^{i}-R_{exp}^{i}}{SD_{exp}^{i}}\right)}^{2}}, \end{array}$$ (10)

where $R_{th}^{i}$ are theoretical values of *R* in Eq (9) and $R_{exp}^{i}$ are experimental values taken from [20]. The index *i* corresponds to different versions of the circuit, wild type and reduced, which are caused by neural eliminations (artificial in the model and by laser ablations in the experiments). $SD_{exp}^{i}$ denotes standard deviations in the experimental values of $R_{exp}^{i}$. The SED function is a measure of similarity of the model to the locomotory behavior of the worm; the smaller its value is the better the fit of the model to the data. Minimization of the above distance with respect to different classes of parameters (see below) produces optimal pattern of synaptic signaling connectome, input, and some intrinsic neurophysiological properties.

### Parameters that are optimized

There are three groups of parameters that we optimize.

- Physiological parameters related to neural activities: gap junction conductance *q*_*e*_, synaptic conductance *q*_*s*_, and the noise amplitude *η* in the input-output function.
- Input coefficients to pre-motor neurons: input vector *σ*_*i*_ (with 6 components taking values either 1 or −1), input amplitude *X*_*o*_, and ASH signaling coefficients *c*_*ash*_ and *f*_*ash*_.
- Matrix *ϵ*_*ij*_ representing the types of synaptic connections, either inhibitory of excitatory. This is a non-symmetric matrix with elements taking values either 0 (if synaptic connection *j* ↦ *i* is inhibitory) or 1 (if the connection is excitatory).

The search ranges of the optimized parameters are given in Table 4.

**Table 4:** Description of optimized parameters.

Variable	Description	Range	Type
*q*_*s*_	synaptic conductance	0–0.07 (mS/cm^2^)	continuous
*q*_*e*_	gap junction conductance	0–0.07 (mS/cm^2^)	continuous
*X*_*o*_	input current amplitude	0–4 (*μ*A/cm^2^)	continuous
*c*_*ash*_	threshold for connection activation	0–2	continuous
*f*_*ash*_	contribution of ASH neuron to input	-1–1	continuous
*η*	voltage noise amplitude	1–10 (mV)	continuous
*σ*_*i*_	vector of the input pattern	1 or −1	discrete
*ϵ*_*ij*_	matrix of synaptic polarities	0 or 1	discrete

### Computational techniques

The software used in this research is in-home written code in Mathematica Wolfram Language and in Java. The special role in the optimization process plays the PCJ (Parallel Computations in Java) library, which enables the massive parallel computations. The Mathematica code was used for prototyping, single point computation analysis and visualization (see Supporting Information). The production code for parameter optimization was written in Java/PCJ (https://github.com/hpdcj/elegans-simulation; https://github.com/hpdcj/evolutionary-algorithm; https://github.com/hpdcj/elegans-optimisation). The Java production code employs the 5(4) Dormand Prince integrator from Apache Commons library [65] as the ODE solver for all differential equations in the model. The genetic algorithm used in the optimisation of our goal function SED is a customary written algorithm based on [66]. The algorithm is composed of the following steps [67]:

1. The set of the 8 parameters used in the model (Table 3) is coded in the form of binary vector (genome).
2. Within the parallel architecture, each computational thread creates a pool of 35 candidate vectors (genomes).
3. Within each computational thread the following loop is performed: for each vector, over all vectors in the pool:
  Mutation—a new parameter vector is created based on three other vectors from the population.
  Crossover—replacement of the selected entries between two vectors. The number of entries to be selected was set to 20%.
  Selection—if the new vector gives a lower goal function, then the new goal function replaces the old one.
4. After 30 executions of this loop on each computational thread, the migration procedure is launched: the best genomes are circulated across all computational threads, and algorithm goes back to point 3.
5. The procedure stops, when the decrease of the goal function does not exceed a given small threshold. The PCJ library permits for performing the distributed memory computation, and scales up to thousands of processor units. We used 1024 threads in our computations, which were performed on a large Haswell cluster located at ICM University of Warsaw. A single job in a production phase can be carried out within an hour of real time.

The computations for the “reshuffled weights” case were conducted in the following way. A reshuffled connectome is the one is which we choose randomly a strength of given connection from 3 possible values (*m*, *m* − *sd*, *m* + *sd*; Fig 2). We draw randomly 100 versions of the connectome and we choose 30 versions with total values of weights that are the closest to the connectome with the mean weights. For these 30 versions of the connectome we perform computations as described in the Results and Methods (see Table 1).

### Numerical values of non-optimized parameters

Values of the the neurophysiological parameters that are not optimized are: *d* = 0.5 *μ*m [43], *V*_*Cl*_ = −50 mV [68], *g*_*L*_ = 0.0067 mS/cm^2^ [20, 43, 59]. Calcium related parameters are *g*_*Ca*_ = 0.043 mS/cm^2^, *g*_*KCa*_ = 0.057 mS/cm^2^, *K*_*D*_ = 30 *μ*M, *τ*_*Ca*_ = 150 msec (all four are motivated by cortical values from [60]; however, in order to keep [Ca^2+^] in a realistic range of 1–100 *μ*M we take *g*_*ca*_ and *g*_*KCa*_ 10 times smaller than in [60], which relates to the fact that *g*_*L*_ for *C. elegans* neurons is also about 10 times smaller than that for cortical neurons). Reversal potentials: *V*_*L*_ = −60 mV, *V*_*Ca*_ = 120 mV, *V*_*K*_ = −90 mV (all three are based on [22, 23] and are believed to be universal [69]). Synaptic transmission characteristics are: *θ*_*ash*_ = −90 mV, *γ*_*ash*_ = 0.03 mV^−1^, *θ*_*i*≠*ash*_ = −40 mV, *γ*_*i*≠*ash*_ = 0.08 mV^−1^, and membrane capacitance *C* = 1 *μ*F/cm^2^ [22, 23].

## Supporting information

S1 Text This file contains Mathematica code for computing and optimizing the goal function SED. (PDF)
