# Supplementary material for: Optimal synaptic signaling connectome for locomotory behavior in Caenorhabditis elegans: Design minimizing energy cost
Source: PLoS Comput Biol. 2017 Nov 20;13(11):e1005834. doi: 10.1371/journal.pcbi.1005834 (PMC5714387; doi:10.1371/journal.pcbi.1005834)
Supplement: S1 Text — (PDF) [file pcbi.1005834.s001.pdf]

Supporting Information for:

„Optimal synaptic signaling connectome for locomotory behavior in C. elegans: design minimizing energy cost”

Franciszek Rakowski[a,b] and Jan Karbowski[c]

[a] *Interdisciplinary Centre for Mathematical and Computational Modeling, University of Warsaw, 02-097 Warsaw, Poland;*
[b] *Mossakowski Medical Research Centre, Polish Academy of Sciences, 02-106 Warsaw, Poland;*
[c] *Institute of Applied Mathematics and Mechanics, Department of Mathematics, Informatics and Mechanics, University of Warsaw, 02-097 Warsaw, Poland.*

```
(*A Mathematica Notebook performing the single point goal function calculation,
for the C.Elegans model.
by F. Rakowski
last mod. 05.04.2017*)


(* Reading sources: *)

srcfiles={"CElegansExperimtalData.m","CElegansPhysMorphData.m","CElegansSo-
lver.m","CElegansUtils.m"};
For[i=1,i<=Length[srcfiles],i++,Get[srcfiles[[i]]]]

Set up parameters:
 mask=prog[.75,∞,connSW];  (*Set up the mask for synnaptic connections*)
pozϵ=Position[mask,1]; (*Position list of supraThreshold synapses *)
dox=3.6 Insert[Tuples[{-1,1},7],0,Flatten[Table[{{n,7},{n,7}},
{n,1,Length[Tuples[{-1,1},7]]}],1]]; (* input combination *)
qqs=0.039;  (* conductivity for chemical sysnapses *)
qqe=0.042;  (* conductivity for gap junctions *)
cASH=0.5; ffASH=-0.8;(* parameters of ASH neuron *)
θθ=prepareθ[hfun1,hfun2,hfun2]; γγ=prepareγ[hfun1,hfun2,hfun2];(* parametres of
the sigmoidal H function *)
ηη=2; (* η *)
headI=32; (* number of the external input variant *)
combNum=1; (* number of the Synaptic type (polarity) variant *)

(* Compute the goal functions: ED and SED: *)

 (* Compute the goal function for the fixed parameter set: the euclidead dis-
tance *)
edGoalFun[mask,qqs, qqe, cASH,ffASH, θθ , γγ , ηη, headI, combNum]
(* Compute the goal function for the fixed parameter set: the standarized eu-
clidead distance *)
sedGoalFun[mask,qqs, qqe, cASH,ffASH, θθ , γγ , ηη, headI, combNum]

(* Graphical comparison of the model with the experimental data. *)

 Needs["ErrorBarPlots`"]
```

```
 CElegansDiff=eFun[diffAllAblations[mask,qqs, qqe, cASH,ffASH, θθ , γγ , ηη,
headI, combNum],ηη];
modelVSexperiment=ListPlot[{CElegansDiff,expTfTbunit,CElegansDiff,expTfTbunit},P
lotRange->{0.2,1.1},Joined->{True,True,False,False},PlotStyle-
>{{Darker[Red],Dashed},{Darker[Blue],Dashed},{Darker[Red],PointSize[Large]},
{Darker[Blue],PointSize[Large]}},Axes->False,Frame->{True,True,False,False},
   FrameTicks->{{All,None},{Table[{i,nlabels[[i]]},{i,1,18}],None}},
   FrameLabel->{"Ablation",Rotate["T_f/(T_f-T_b)",-90 Degree]},LabelStyle-
>{Bold,Black,12},PlotLegends->{None,None,"model","experiment"}];
 Show[modelVSexperiment,ErrorListPlot[{expTfTbunit,errTfTbunit}//Transpose]]
```

(* L I B R A R I E S*)

(* Mathematica package CElegansSolver.m
by F. Rakowski.
last mod. 05.04.2017 *)

```
(* Definition of ODE's, and goal functions*)

(* Auxiliary functions and function declarations*)
funH[x_,\[Gamma]_,\[Theta]_]:=1/(1+E^(-\[Gamma](x-\[Theta])));
m[i_]:=1/(1+E^(-(fn[[i]]+20)/9));
citer[j_]:=Table[i,{i,Drop[{1,2,3,4,5,6,7,8,9},{j}]}];
fn={ash[t],ava[t],avb[t],avd[t],ave[t],dva[t],eb[t],ef[t],pvc[t],x[t]};
ca={cash[t],cava[t],cavb[t],cavd[t],cave[t],cdva[t],ceb[t],cef[t],cpvc[t]};

ur[nonZeroSW_, nonZeroGJ_, a_, \[Epsilon]_,x_,physParam_,physVarParam_]:=
 Module[{eqnsVolMotN, eqnsVolIntN, eqnsVol, eqnsCon, initialsVol,
   initialsCon},
  (* Routine creating the ODE's
    nonZeroSW - synaptic weights,
    nonZeroGJ - gap junction weights,
    a - ablation vector,
    \[Epsilon] - synaptic type: 0 - inhibitory, 1 - excitatory
    x - input vector
  *)

  eqnsVolIntN =
   Table[cC D[fn[[i]], t] == -gL (fn[[i]] - vl) -
      gCa m[i]^2 (fn[[i]] - vCa) -
      gKCa ca[[i]]/(kD + ca[[i]]) (fn[[i]] - vK) -
      Sum[a[[j]] nonZeroSW[[i, j]] funH[
         fn[[j]], (\[Gamma]/.physVarParam)[[j]], (\[Theta]/.physVarParam)[[j]]]
(fn[[i]] - (1 - \[Epsilon][[i,
              j]]) vCl), {j, citer[i]}]
      - Sum[
       a[[i]] a[[j]] nonZeroGJ[[i, j]] (fn[[i]] - fn[[j]]), {j,
        citer[i]}] + x[[i]](1 + a[[1]] fASH funH[fn[[1]],(\[Gamma]/.physVarPa-
ram)[[1]],(\[Theta]/.physVarParam)[[1]]]), {i, {2, 3, 4, 5, 6, 9}}];

  eqnsVolMotN =
   Table[cC D[fn[[i]], t] == -gL (fn[[i]] - vl) -
      Sum[a[[j]] nonZeroSW[[i, j]] funH[
         fn[[j]], (\[Gamma]/.physVarParam)[[j]], (\[Theta]/.physVarParam)[[j]]]
(fn[[i]] - (1 - \[Epsilon][[i,
              j]]) vCl), {j, citer[i]}] -
      Sum[a[[i]] a[[j]] nonZeroGJ[[i, j]] (fn[[i]] - fn[[j]]), {j,
        citer[i]}], {i, {7, 8}}];

  eqnsVol =
   Flatten[{eqnsVolIntN[[1 ;; 5]], eqnsVolMotN, eqnsVolIntN[[6]]}];
  initialsVol = Table[(fn[[i]] /. t -> 0) == 2, {i, 2, 9}];
```

```
  eqnsCon =
   Table[D[ca[[i]], t] == -ca[[i]]/tauCa -
      2 gCa (m[i]^2) /(d far) (fn[[i]] - vCa), {i, {2, 3, 4, 5, 6,
      9}}];
  initialsCon =
   Table[(ca[[i]] /. t -> 0) == 2, {i, {2, 3, 4, 5, 6, 9}}];

  eqnini = Join[eqnsVol, eqnsCon, initialsVol, initialsCon];
  eqnini/.Join[physParam,physVarParam]
  ]

efeb[nonZeroSW_, nonZeroGJ_, a_, \[Epsilon]_,x_,physParam_,physVarParam_] :=
 Module[{solutionBDF, feb, fef},
  solutionBDF =
  NDSolve[ur[nonZeroSW, nonZeroGJ, a, \[Epsilon],x,physParam,physVarParam],
   Join[fn[[2 ;; 9]], ca[[{2, 3, 4, 5, 6, 9}]]], {t, 0, 4000},
   Method -> {"BDF"}];

  feb = (eb[t] /. solutionBDF[[1, 6]]) /. t -> 3000;
  fef = (ef[t] /. solutionBDF[[1, 7]]) /. t -> 3000;
  {fef, feb}

  ]
  diffSingleAblation[nonZeroSW_, nonZeroGJ_, a_, \[Epsilon]_,x_,physParam_,phys-
VarParam_] :=
 Module[{solutionBDF, feb, fef},
  solutionBDF =
  NDSolve[ur[nonZeroSW, nonZeroGJ, a, \[Epsilon],x,physParam,physVarParam],
   Join[fn[[2 ;; 9]], ca[[{2, 3, 4, 5, 6, 9}]]], {t, 0, 4000},
   Method -> {"BDF"}];
  feb = (eb[t] /. solutionBDF[[1, 6]]) /. t -> 3000;
  fef = (ef[t] /. solutionBDF[[1, 7]]) /. t -> 3000;
  fef-feb
  ]
  (* Euclidean distance goal function *)
 edGoalFun::usage =
  "arg: maska, qs, qe, ashCoeff,fASH, \[Theta], \[Gamma],  \[Eta], HeadInput \
Number,  SynnapticCombination Number";
edGoalFun[mask_,qs_?NumberQ, qe_?NumberQ, ashCoeff_?NumberQ, tmpfASH_?NumberQ,
tmp\[Theta]_, tmp\[Gamma]_, \[Eta]_?NumberQ, k_?NumberQ,
  l_?NumberQ] := Module[{CSW, CGJ, tmpPar, kk},
  (*arg: mask - maska connectomu, qs,qe, ashCoeff,\[Theta],\[Gamma],No of input
Comb,
  No of synnaptic Comb,\[Eta]*)
  CSW = connSW qs mask;
  CGJ = connGJ qe;
  tmpPar = {\[Gamma] -> tmp\[Gamma], \[Theta] -> tmp\[Theta],
    ash[t] -> ashCoeff * tmp\[Theta][[1]], fASH -> tmpfASH};

  kk = Table[
    diffSingleAblation[CSW, CGJ, abl[[i]], polarMatrixSubst[l], dox[[k]], phys-
Param,
     tmpPar], {i, 1, Length[abl]}];
  compTfTb[kk, \[Eta]]
  ]
  (* Standarized Euclidean distance goal function *)
  sedGoalFun[mask_,qs_?NumberQ, qe_?NumberQ, ashCoeff_?NumberQ,tmpfASH_?NumberQ,
tmp\[Theta]_, tmp\[Gamma]_, \[Eta]_?NumberQ, k_?NumberQ,
  l_?NumberQ] := Module[{CSW, CGJ, tmpPar, kk},
  (*arg: mask - maska connectomu, qs,qe, ashCoeff,\[Theta],\[Gamma],No of input
Comb,
  No of synnaptic Comb,\[Eta]*)
  CSW = connSW qs mask;
  CGJ = connGJ qe;
```

```
  tmpPar = {\[Gamma] -> tmp\[Gamma], \[Theta] -> tmp\[Theta],
    ash[t] -> ashCoeff * tmp\[Theta][[1]],fASH -> tmpfASH};

  kk = Table[
    diffSingleAblation[CSW, CGJ, abl[[i]], polarMatrixSubst[l], dox[[k]], phys-
Param,
     tmpPar], {i, 1, Length[abl]}];
     sed[eFun[kk,\[Eta]],errTfTbunit,expTfTbunit]
  ]

  diffAllAblations[mask_,qs_?NumberQ, qe_?NumberQ, ashCoeff_?NumberQ,tmpfASH_?
NumberQ, tmp\[Theta]_, tmp\[Gamma]_, \[Eta]_?NumberQ, k_?NumberQ,
  l_?NumberQ] := Module[{CSW, CGJ, tmpPar, kk},
  (*arg: mask - maska connectomu, qs,qe, ashCoeff,\[Theta],\[Gamma],No of input
Comb,
  No of synnaptic Comb,\[Eta]*)
  CSW = connSW qs mask;
  CGJ = connGJ qe;
  tmpPar = {\[Gamma] -> tmp\[Gamma], \[Theta] -> tmp\[Theta],
    ash[t] -> ashCoeff * tmp\[Theta][[1]],fASH -> tmpfASH};

  kk = Table[
    diffSingleAblation[CSW, CGJ, abl[[i]], polarMatrixSubst[l], dox[[k]], phys-
Param,
     tmpPar], {i, 1, Length[abl]}]
  ]
```

```
(* Mathematica package CElegansPhysMorphData.m
    by F. Rakowski.
    last mod. 05.04.2017 *)

(* PHYSIOLOGICAL CONSTANTS *)
physParam= {
cC ->1, (*uF/cm^2 Capacitance of the membrane*)
gL -> 0.0067, (*mS/cm^2 leak conductance*)
vl -> -60, (*mV leak reversal potential*)
gCa -> 0.043, (*mS/cm^2 Calcium conductance*)
vCa -> 120 ,(*mV Calcium reversal potential*)
gKCa -> 0.057, (*mS/cm^2 Potasium-Ca gated conductance*)
vK -> -90,(*mV Potasium reversal potential*)
kD -> 30, (*uM Calcium concentration*)
vCl -> -50,(*mV Chloride reversal potential*)
tauCa -> 150, (*ms - relaxation time for Ca concentration*)
far -> 9.648, (*10 mC/umol = 10 kC/mol Faraday constant*)
d -> 0.5 (*um *) }

(*CONNECTIVITY MATRIX*)
(* Numerical values of synaptic connectivity matrix *)
connSW = {{0, 0, 0, 0, 0, 0, 0, 0, 0}, {1.75, 0, 6.75, 15.75, 10.5, 2., 0.25,
  0, 5.}, {2.25, 0.5, 0, 0.25, 0, 0.5, 0, 0, 7.75}, {3., 1., 0.75, 0,
  0.25, 0, 0.25, 0, 3.25}, {0.75, 1., 0.75, 0, 0, 7., 0, 0, 1.25}, {0,
   0, 0, 0, 0, 0, 0, 0.5, 2.}, {0, 41.75, 1.5, 7., 8.25, 1., 0, 0,
  1.}, {0, 2.5, 0.25, 0.25, 0.25, 6.5, 0, 0, 12.}, {0, 7., 0, 0.25,
  0.25, 2., 1.25, 0.25, 0}};

(* Numerical values of gap junction connectivity matrix *)
  connGJ = {{0, 0, 0, 0, 0, 0, 0, 0, 0}, {0, 0, 0, 0, 0, 0, 25.5, 3.5, 2.5}, {0,
  0, 0, 0, 0, 1., 0.5, 13.75, 0}, {0, 0, 0, 0, 0, 0, 0, 0, 0}, {0, 0,
  0, 0, 0, 0, 0, 0, 0}, {0, 0, 1., 0, 0, 0, 0, 0.5, 0.5}, {0, 25.5,
  0.5, 0, 0, 0, 0, 0, 0.75}, {0, 3.5, 13.75, 0, 0, 0.5, 0, 0,
```

```
  0.75}, {0, 2.5, 0, 0, 0, 0.5, 0.75, 0.75, 0}};

  (* Logical presence of connections*)
BINconnSW = connSW /. x_ /; x != 0 -> 1;
BINconnGJ = connGJ /. x_ /; x != 0 -> 1;

(*Synaptic polarity Matrix*)
\[Epsilon] = BINconnSW /. x_ /; x == 0 -> False

(* Sigmoidal function parameteres *)
hfun1={0.08,-70}; (*for ASH neuron*)
hfun2={0.08,-40}; (*for Interneurons and Motoneurons*)
```

```
(* Mathematica package CElegansExperimentalData.m
    by F. Rakowski.
    last mod. 05.04.2017 *)

(* Matrix of all ablations used in the experiment *)
ablMatrix = {{1, 1, 1, 1, 1, 1, 1}, {0, 1, 1, 1, 1, 1, 1}, {1, 0, 1, 1, 1, 1,
1},
   {1, 1, 0, 1, 1, 1, 1}, {1, 1, 1, 0, 1, 1, 1}, {1, 1, 1, 1, 1, 0, 1},
   {1, 1, 1, 1, 1, 1, 0}, {0, 0, 1, 1, 1, 1, 1}, {0, 1, 0, 1, 1, 1, 1},
   {0, 0, 0, 1, 1, 1, 1}, {1, 0, 0, 1, 1, 1, 1}, {1, 0, 1, 1, 1, 1, 0},
   {1, 1, 0, 1, 1, 1, 0}, {1, 0, 0, 1, 1, 1, 0}, {1, 1, 0, 0, 1, 1, 0},
   {1, 1, 0, 1, 1, 0, 0}, {1, 0, 0, 1, 0, 1, 0}, {1, 1, 1, 1, 1, 0, 0}};

(* Definition of the ablation vector *)
abl = Table[
   Flatten[{ablMatrix[[k, 1 ;; 6]], 1, 1, ablMatrix[[k, 7]]}], {k, 1,
    18}];
(* Experimental data {Tforward, Tbackward}*)
expData = {{7.82, 2.53}, {12.05, 0.95}, {0.71, 0.53}, {2.26, 2.14}, {4.24,
  3.12}, {1.51, 1.23}, {8.2, 1.7}, {1.91, 0.85}, {2.05, 2.04}, {0.75,
  0.52}, {0.56, 0.46}, {4.09, 0.67}, {0.91, 1.19}, {0.93,
  0.47}, {1.33, 0.94}, {2.04, 1.29}, {0.6, 0.39}, {2.18, 1.35}};

(* Ratio: *)
expTfTb = {3.09091, 12.6842, 1.33962, 1.05607, 1.35897, 1.22764, 4.82353, \
2.24706, 1.0049, 1.44231, 1.21739, 6.10448, 0.764706, 1.97872, \
1.41489, 1.5814, 1.53846, 1.61481};

(* Normalised Ratio:*)
expTfTbunit = {0.755556, 0.926923, 0.572581, 0.513636, 0.576087, 0.551095, \
0.828283, 0.692029, 0.501222, 0.590551, 0.54902, 0.859244, 0.433333, \
0.664286, 0.585903, 0.612613, 0.606061, 0.617564};

(* Error for normalised ratio: *)
errTfTbunit={0.0331723,0.0215089,0.0494927,0.071064,0.131853,0.0455805,0.0640241
,\
0.0970122,0.110012,0.0521812,0.103968,0.0367469,0.169268,0.0941945,0.128526, \
0.136071,0.0901046,0.0583286};
```

```
(* Mathematica package CElegansUtils.m
   by F. Rakowski,
   last mod. 5.04.2017    *)

(* Combinatorial Tools *)
```

```
fdo\[Epsilon][l_,dlugosc_]:=PadLeft[IntegerDigits[l-1,2],dlugosc]
(* fdo\[Epsilon] generates l-th variant of synaptic polarity *)

prog[zd_, zg_, a_] := Module[{w1, w2, dl},
  (* aux function: marking by ones all entries of the connectivity matrix,
wchich values are between
  zd and zg. *)
  dl = Length[a];
  w1 = Array[
    a[[#1, #2]] /. x_ /; (x > zd && x <= zg) -> (-10) &, {dl, dl}];
  w2 = Array[w1[[#1, #2]] /. x_ /; x > (-10) -> 0 &, {dl, dl}]/(-10)
  ]

  polarMatrixSubst[k_] := Module[{co,zerosE},
  (*substitutes the wild type polarity matrix {Subscript[\[Epsilon], i,j]} by
the matrix representig k-th
  variant of all possible polarity variants  *)
  zerosE = ConstantArray[0, {9, 9}];
  co = Table[poz\[Epsilon][[i]] -> fdo\[Epsilon][k,Length[poz\[Epsilon]]][[i]],
{i, 1, Length[poz\[Epsilon]]}];
  zerosE=ReplacePart[zerosE, co];
  zerosE[[All,7;;8]]=ConstantArray[1,{9,2}];
  zerosE
  ]

(*Auxiliary functions*)

eFun[pW_, \[Eta]_] := E^(pW/\[Eta])/(1 + E^(pW/\[Eta]));
sed[vDane_,vErr_,vProbe_]:=Sqrt[Total[((vDane-vProbe)/vErr)^2]]

compTfTb[pW_, \[Eta]_] :=
  EuclideanDistance[eFun[pW, \[Eta]], expTfTbunit];

prepare\[Gamma][ash_,int_,mot_]:=First/@{ash,int,int,int,int,int,mot,mot,int};
prepare\[Theta][ash_,int_,mot_]:=Last/@{ash,int,int,int,int,int,mot,mot,int};

(* Auxiliary visualisation functions *)
nlabelsAux=Prepend[Table[StringReplace[ToString[DeleteCases[-(abl[[i]]-
1)*fn[[;;9]],0]],"[t]"->""]//ToUpperCase,{i,2,18}],{"WILD"}];
nlabels=Table[Rotate[StringReplace[nlabelsAux[[i]]//ToString,{", "->"-","{"-
>"","}"->""}],90 Degree],{i,1,18}];
```
